# Proanthocyanidin synthesis in *Theobroma cacao*: genes encoding anthocyanidin synthase, anthocyanidin reductase, and leucoanthocyanidin reductase

**DOI:** 10.1186/1471-2229-13-202

**Published:** 2013-12-05

**Authors:** Yi Liu, Zi Shi, Siela Maximova, Mark J Payne, Mark J Guiltinan

**Affiliations:** 1 Huck Institute of Life Sciences, The Pennsylvania State University, University Park, PA 16802, USA; 2 Department of Plant Science, The Pennsylvania State University, 422 Life Sciences Building, University Park, PA 16802, USA; 3 Hershey Center for Health and Nutrition, The Hershey Company, 1025 Reese Ave, Hershey, PA 17033, USA; 4 Present address: Cellular & Molecular Pharmacology, Mission Bay Campus, Genentech Hall, University of California, San Francisco, N582/Box 2280, 600 16th Street, San Francisco, CA 94158, USA

## Abstract

**Background:**

The proanthocyanidins (PAs), a subgroup of flavonoids, accumulate to levels of approximately 10% total dry weight of cacao seeds. PAs have been associated with human health benefits and also play important roles in pest and disease defense throughout the plant.

**Results:**

To dissect the genetic basis of PA biosynthetic pathway in cacao (*Theobroma cacao*), we have isolated three genes encoding key PA synthesis enzymes, anthocyanidin synthase (ANS), anthocyanidin reductase (ANR) and leucoanthocyanidin reductase (LAR). We measured the expression levels of *TcANR*, *TcANS* and *TcLAR* and PA content in cacao leaves, flowers, pod exocarp and seeds. In all tissues examined, all three genes were abundantly expressed and well correlated with PA accumulation levels, suggesting their active roles in PA synthesis. Overexpression of *TcANR* in an Arabidopsis *ban* mutant complemented the PA deficient phenotype in seeds and resulted in reduced anthocyanidin levels in hypocotyls. Overexpression of *TcANS* in tobacco resulted in increased content of both anthocyanidins and PAs in flower petals. Overexpression of *TcANS* in an Arabidopsis *ldox* mutant complemented its PA deficient phenotype in seeds. Recombinant TcLAR protein converted leucoanthocyanidin to catechin *in vitro*. Transgenic tobacco overexpressing *TcLAR* had decreased amounts of anthocyanidins and increased PAs. Overexpressing *TcLAR* in Arabidopsis *ldox* mutant also resulted in elevated synthesis of not only catechin but also epicatechin.

**Conclusion:**

Our results confirm the *in vivo* function of cacao *ANS* and *ANR* predicted based on sequence homology to previously characterized enzymes from other species. In addition, our results provide a clear functional analysis of a *LAR* gene *in vivo*.

## Background

Flavonoids are a diverse group of plant secondary metabolites with various biological functions that play important roles during plant development. They are involved in plant defenses against insects, pathogens and microbes, in absorption of free radicals and UV light, and in attraction of beneficial symbionts and pollinators [1-4]. Proanthocyanidins (PAs, also known as condensed tannins) are components of metabolites synthesized through the general flavonoid pathway. Their main known function is to provide protection against microbial pathogens and invasions of insects and herbivores through multiple mechanisms [1]. PAs have metal chelating activity that results in severe limitation of bacterial growth; PAs can also associate with and irreversibly precipitate proteins, which is responsible for the astringent taste that repels herbivores. Furthermore, PAs can be oxidized to quinones which not only are powerful antibiotics themselves, but also can initiate cross-linking of cell walls to increase the strength of this physical barrier to pathogens [5-8]. In addition to these functional roles in plants, PAs, especially those from cacao, have recently been suggested to be beneficial to humans by improving cardiovascular health through activation of nitric oxide synthase, by providing cancer chemopreventative effects, and also through neuroprotective activities [9-11].

**Figure 1:**
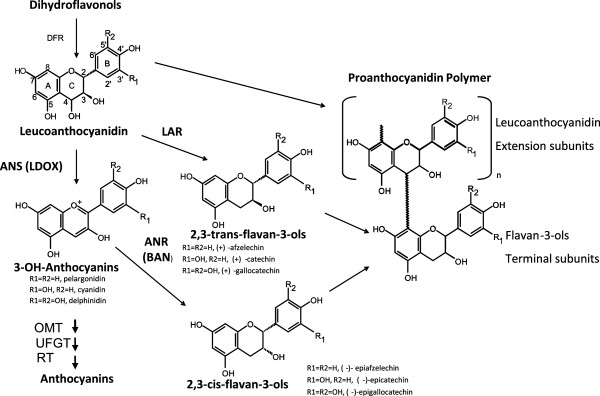
**Outline of the proanthocyanidin synthesis pathway (adapted from [**[17]**]).** Enzymes are represented in uppercase letters. DFR, dihydroflavonol 4-reductase, EC 1.1.1.219; ANS, anthocyanidin synthase, EC 1.14.11.19; ANR, anthocyanidin reductase, EC 1.3.1.77; LAR, leucoanthocyanidin reductase, EC 1.17.1.3; OMT, O-methyltransferases, E.C.2.1.1.-; UFGT, UDP-glucose: anthocyanidin/flavonol 3-O-glucosyltransferase, EC 2.4.1.115; RT, rhamnosyltransferase, EC 2.4.1.159.

The biosynthetic pathways that lead to the production of flavan-3-ols ((+)-catechin and (−)-epicatechin), the building blocks of PAs, have been well studied in the model plant species maize (*Zea mays*) and Arabidopsis as summarized in Figure 1[12,13]. Biosynthesis of flavan-3-ols involves three principal enzymes: leucoanthocyanidin reductase (LAR), anthocyanidin synthase (ANS; also called leucoanthocyanidin dioxygenase, LDOX), and anthocyanidin reductase (ANR; in Arabidopsis, the product of *BANYULS* gene). The synthesis of PAs and anthocyanins share common steps leading to flavan-3,4-diols (such as leucoanthocyanidin), which can be converted to catechin (2,3-*trans*-flavan-3-ol) by LAR [14] or to anthocyanidin by ANS [15,16]. Anthocyanidin then either serves as the substrate for the synthesis of epicatechin (2,3-*cis*-flavan-3-ol) by ANR [17], or can otherwise be converted to anthocyanin by glycosylation [18]. Both catechin and epicatechin act as the initiators for PA polymerization, with intermediates derived from leucoanthocyanidin, catechin or epicatechin added sequentially as extension units [1]. However, the details of the polymerization process are unclear and it is not known whether this is a spontaneous or an enzyme-catalyzed reaction. Recent work has identified two new enzymes downstream of flavan-3-ols that are involved in key steps of PA polymer biosynthesis, an epicatechin 3’-O-glucosyltransferase in *Medicago truncatula*[19] and MATE transporters from Arabidopsis and *Medicago truncatula*[20] that transport epicatechin 3’-*O*-glucoside to the vacuole in which it is likely that PA polymerization occurs [21].

*ANS* and *ANR* genes have been biochemically and genetically characterized in Arabidopsis [15,17,22,23], *Medicago truncatula*[17,24] and *Vitis vinifera* (grape) [25]. The Arabidopsis *ans* (*ldox*) mutant exhibits a deficiency in both anthocyanin accumulation in hypocotyls and PA deposition in seeds that results in a transparent testa phenotype [15]. Seeds of the Arabidopsis *banyuls* (*anr*) mutant exhibit a lack of PAs and a hyper-accumulation of anthocyanins, resulting in a dark red color reminiscent of the famous Banyuls wine produced in southern France [22]. Over-expression of *ANR* genes from *Medicago* and grape in tobacco results in a loss of anthocyanin pigments in flower petals and elevated levels of PAs [17,25]. Antisense down-regulation of *ANS* in *Medicago* results in reduction of both anthocyanins in leaves and PAs in seeds [24].

*LAR* genes have been isolated from various plant species including *Desmodium uncinatum*[14], *Vitis vinifera*[25], *Lotus corniculatus*[26] and *Medicago truncatula*[24] and their corresponding protein functions have been characterized by *in vitro* recombinant enzyme assays. However, the genetic evidence for *LAR* function is rather indirect and less convincing than it is for *ANR* and *ANS*, as discussed by Pang et al. [24]. It appears that the genomic sequence of *Arabidopsis thaliana* does not contain an intact *LAR* orthologue, and correspondingly, catechin is not detected in Arabidopsis seed extracts [12,14,15]. *LAR* genes are expressed in other plant species that accumulate not only epicatechin but also catechin [14,25,26]. For example, grape and *Lotus* express both *LAR* and *ANR* genes and synthesize PAs consisting of both catechin and epicatechin [25-27]. In *Medicago*, although both *LAR* and *ANR* are expressed, the PAs are composed almost entirely of epicatechin [24].

In Arabidopsis and *Medicago*, PA accumulation and gene expression is quantitatively and spatially limited to seed coats, making it remarkably difficult for biochemical analysis [12,24]. In contrast, *Theobroma cacao* (*Tc*) produces significant amounts of PAs in various tissues including leaves and beans; up to 12% of dry weight in leaves [28] and approximately 10% in mature beans [29]. Furthermore, large amounts of catechin and epicatechin monomers as well as their related polymers of different lengths have been detected in cocoa powder [28,30]. Considering the wide range of health benefits suggested for PAs and its significance for plant resistance, we targeted this pathway for molecular-genetic analysis.

This manuscript describes the isolation and expression of the *TcANR*, *TcANS*, and *TcLAR* genes encoding the key enzymes in proanthocyanidin biosynthesis. We measured PA content in different cacao tissues and performed functional characterization of the *TcANR*, *TcANS*, and *TcLAR* gene products through *in vivo* tests. The results presented here provide background and genetic tools that will be useful in the development of new cacao varieties with altered PA profiles.

## Results

### Molecular cloning and sequence analysis of TcANR, TcANS and TcLAR genes

The general pathway for the synthesis of proanthocyanidin and anthocyanins indicates the key enzymes ANR, ANS and LAR that carry out the biochemical steps at a critical metabolic branch point (Figure 1). To explore the genetic control of this important pathway in cacao, putative *TcANR*, *TcANS* and *TcLAR* cDNA sequences were identified in a collection of *Theobroma cacao* expressed sequence tags (ESTs) [31] by querying the cacao ESTtik database (http://esttik.cirad.fr/) with protein sequences of Arabidopsis *BANYULS* (NP_176365), Arabidopsis *LDOX* (Q96323) and *Desmodium LAR* (CAD79341). ESTs similar to each gene were assembled into contigs to determine consensus full-length open reading frames (ORF) by alignment with cDNAs of homologous genes from other species and predictions from the ORF Finder program (http://www.ncbi.nlm.nih.gov/projects/gorf/). Full-length cDNAs of each gene were amplified by reverse transcription-PCR (RT-PCR) using RNA isolated from young leaves of cacao (Scavina 6). The *TcANR* cDNA (GU324348) contained a 1,008-bp open reading frame (ORF) encoding a protein of 336 amino acids that showed a 63% identity with the Arabidopsis *BANYULS* gene at the amino acid level. The *TcANS* cDNA (GU324350) contained an ORF of 1,062-bp, which encodes a protein of 354 amino acids with 82% amino acid identity with the Arabidopsis *LDOX* gene. The *TcLAR* cDNA (GU324352) contained a 1,083-bp ORF encoding a protein of 361 amino acids with 61% amino acid identity with the *Desmodium* LAR protein.

**Figure 2:**
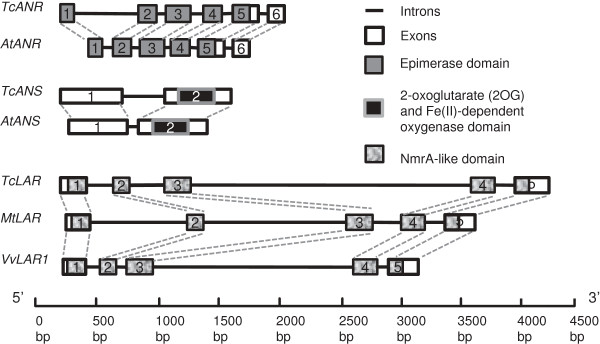
**Structures of cacao *TcANR, TcANS *and *TcLAR *genes with predicted protein domains, and comparison with their orthologues from Arabidopsis (*AtANR *and *AtANS*), *Medicago *(*MtLAR*) *and Vitis *(*VvLAR*).** Green boxes represent exons and lines introns. Lengths in base pairs are indicated by the scale at the bottom.

To determine the genomic sequences of the putative cacao *ANS*, *ANR* and *LAR* genes, the cDNAs were used to screen a cacao BAC library by hybridization. A portion of each hybridizing BAC clone was sequenced using primers designed from the corresponding cDNAs. The genomic structure of each gene was established by alignment with its cDNA sequence (Figure 2). We also retrieved gene models of the *ANS* and *ANR* gene from Arabidopsis (AT4G22880 and AT1G61720) and *LAR* from *Medicago* and grape (BN000703 and NC_012007 c2622652-2619277) and compared them with the corresponding cacao genes.

The coding region of *TcANR* (GU324347) consisted of 6 exons and 5 introns distributed over 2,005-bp. The coding region of *TcANS* (GU324349) was shorter, having only 1,418-bp and consisting of 2 exons and 1 intron. The genomic organizations of these two cacao genes were nearly identical to the corresponding Arabidopsis genes, which had the same exon and intron numbers. Moreover, the lengths of the exons were very similar between Arabidopsis and cacao: for *ANR*, the numbers of nucleotides were precisely the same for the exons 2, 3, 4, and 5, although the lengths of introns were more variable.

Since *LAR* does not have an orthologue in Arabidopsis, we compared the genomic organization of *TcLAR* with *MtLAR* and *VvLAR* (Figure 2). The genomic organization of the coding region of *TcLAR* (GU324351) was similar to both *MtLAR* and *VvLAR,* consisting of 5 exons and 4 introns. As observed with the *ANR* gene, the middle three exons (exon 2, 3 and 4) of *LAR* from all three species had identical lengths. *TcLAR* exhibited an extremely long third intron of 2,338 bp; similarly, *VvLAR* also featured a long third intron of 1,661 bp, while *MtLAR* contained two long introns (intron 1 and intron 2) that are 812-bp and 1,178-bp respectively.

The analysis of the complete cacao genome sequence of the criollo variety was consistent with these results and allowed us to localize the position of each gene relative to the molecular-genetic map [32]. The *ANR* gene was located on chromosome 6 (Tc06_g018030, GenBank: GU324347.1) and was 99.75 identical to the cDNA described above. Similarly, a single *ANS* gene was located on chromosome 3 (Tc03_g026420, GenBank: GU324349.1) and shared 99.3% identity to the cDNA described above. The *LAR* gene was also located on chromosome 3 Tc03_g002450, GenBank: GU324351.1) and shares 98.5% identity to its corresponding cDNA. However, two more distantly related genes were identified in the cacao genome assembly that were not identified in the cacao EST collection. One gene (Tc05_p002410) encodes a protein that shares only 37% identity and 57% similarity with the TcANS protein and a second (Tc02_g034610) shares 63% identity and 76% similarity with the TcLAR protein. For this study, we focused our further work on the three genes of highest similarity to the well-characterized corresponding Arabidopsis and *Desmodium* genes.

**Figure 3:**
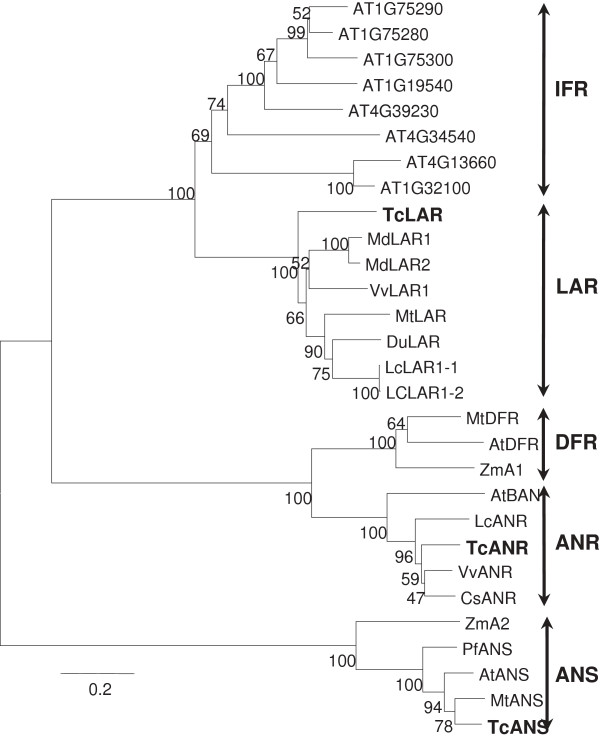
**Phylogenetic analysis of the LAR, ANS and ANR proteins as well as related IFR and DFR proteins of the RED superfamily.** The protein sequences were aligned as described in Material and Methods. The scale bar represents 0.2 substitutions per site. Numbers indicate bootstrap values. The tree includes LAR, ANR and ANS proteins from cacao, which are shown in bold font, as well as from other species whose enzymatic activities have been shown in previous publications. Also included in the tree are 8 IFR like proteins from Arabidopsis (labeled with their locus numbers). All the other proteins are labeled by the species they come from followed by their catalytic activities. The species represented and their GenBank accession numbers in the LAR group are *Medicago* (MtLAR, CAI56327), *Vitis vinifera* (VvLAR, CAI26309), *Desmodium uncinatum* (DuLAR, CAD79341), *Malus domestica* (MdLAR1, Q5D7Y1; MdLAR2, Q5D7Y2) and *Lotus corniculatus* (LcLAR1-1, ABC71324; LcLAR1-2, ABC71325); in the DFR group are *Zea mays* (ZmA1, CAA28734), *Arabidopsis thaliana* (AtDFR, NP_199094) and *Medicago truncatula* (MtDFR, AAR27014); in the ANR group are *Arabidopsis thaliana* (BAN, NP_176365), *Medicago truncatula* (MtANR, AAN77735), *Vitis vinifera* (VvANR, CAD91911), *Camellia sinensis* (CsANR, AAT68773) and *Lotus corniculatus* (LcANR, ABC71337); and in the ANS group are *Perilla frutescens* (PfANS, O04274), *Arabidopsis thaliana* (AtANS, Q96323), *Zea mays* (ZmA2, CAA39022) and *Medicago truncatula* (MtANS, ABU40983). The sequence alignment used to generate this figure can be found in “Additional file 1: Figure S1”.

LAR and ANR proteins belong to the reductase-epimerase-dehydrogenase (RED) superfamily, although their relationships are rather distant. ANS belongs to a different protein superfamily, the 2-oxoglutarate-dependent dioxygenase (2-ODD) superfamily, although it shares the same substrate with LAR (Figure 1). A phylogenetic tree was constructed using the neighbor-joining method with the sequences of functionally-tested proteins of LAR, ANR, ANS and DFR from various plant species. The tree construction also included all the IFR-like proteins from Arabidopsis that are most closely related to LAR proteins (Figure 3, see protein alignment in Additional file 1: Figure S1). The tree was noticeably bifurcated into two clades: all RED proteins (including ANR, LAR, DFR and IFR) constituting one clade, and all ANS proteins constituting the other. Within the RED superfamily clade, the IFR, LAR, ANR and DFR proteins are clearly divided into four distinct groups with IFR and LAR forming a subgroup that is distantly related to the subgroup formed by DFR and ANR groups. The cacao ANR, ANS and LAR proteins used in the current research all clustered within their own groups.

### Expression profiles of TcANR, TcANS and TcLAR genes

**Figure 4:**
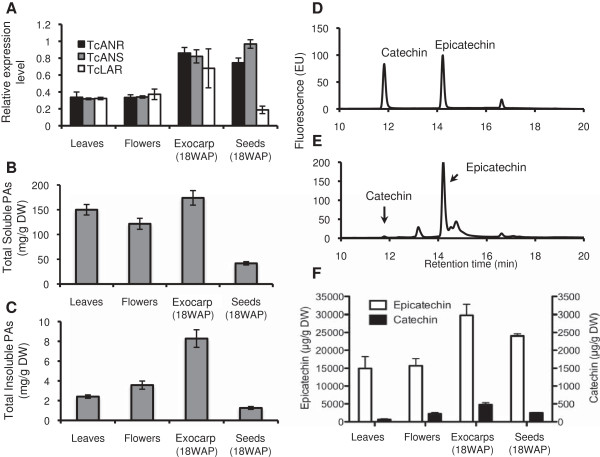
**Expression analysis of key PA biosynthesis genes, and PA composition in various tissues of *Theobroma cacao*. A**, Gene expression levels of *TcANR*, *TcLAR1* and *TcLAR1*. Expression was determined by semi-quantitative RT-PCR normalized relative to the expression of *TcActin* in each sample. **B**, Levels of soluble PAs expressed as mg PA per g of dry weight. **C**, Levels of insoluble PAs expressed as mg PA per g of dry weight. **D**, Catechin and epicatechin standards analyzed by HPLC. **E**, Representative HPLC analysis of flavan-3-ols extracted from cacao leaves. **F**, Flavan-3-ol accumulation and composition analyzed by HPLC. All data are presented as means ± SE, for gene expression data, n ≥ 3, for PA level data, n ≥ 5. DW, dry weight.

To assess the involvement of *TcANR, TcANS* and *TcLAR* in PA biosynthesis in cacao, transcript levels were investigated in various tissues including leaves, flowers and pod tissues (Figure 4A, tissue definition and collection are described in Methods). Gene transcripts levels were assessed by semi-quantitative RT-PCR. *TcActin* was chosen as a reference to normalize gene expression because both cacao microarray analyses (Z. Shi and S. Maximova, unpublished data) and data from this study suggested a relatively constant spatial and temporal expression of this gene for all cacao tissues examined. PA synthesis genes, *TcANR, TcANS* and *TcLAR* were expressed in all tissues examined, with relatively higher expression levels in pod exocarp and seeds and lower expression in leaves and flowers. Moreover, their expression levels were relatively similar within each tissue except seeds, in which the expression level of *TcLAR* was much lower than *TcANR* and *TcANS.*

### PA levels in various cacao tissues

To determine the concentrations of PAs in different cacao tissues, samples were first extracted to obtain a soluble PA fraction. The residues left after soluble PA extraction were assayed using butanol-HCl (Yu-Guang [33]) to measure the amount of insoluble PAs represented as larger polymers. Because insoluble PA polymers will crystallize and bind to proteins and cell wall components, this interference may reduce the extraction efficiency of the insoluble PAs [24,34]. As a result, comparing the relative amount of these two fractions within the same tissue is difficult. However, the accumulation pattern of each fraction is comparable among different tissues. High levels of both soluble and insoluble PAs were detected in all tissues examined, ranging from approximately 40 mg/g DW in seeds to more than 170 mg/g DW in exocarp for soluble PAs (Figure 4B) and ranging from approximately 1.2 mg/g DW in seeds to 8 mg/g DW in exocarps for insoluble PAs (Figure 4C). The accumulation of both soluble and insoluble PAs were in good correlation with the expression patterns of PA synthesis genes, both of which showed highest levels in fruit exocarp. However, both soluble and insoluble PA levels were much lower in seeds than in other tissues, which correlated with *TcLAR* but not *TcANR* and *TcANS*.

To determine the composition of monomer PAs in cacao, soluble PAs extracts prepared from all four tissues were further separated and quantified by HPLC. The data are presented for catechin and epicatechin concentrations as μg of catechin or epicatechin per g of dry tissue (Figure 4D-F). PAs consisted almost entirely of epicatechin with less than 2% catechin. In all tissues examined, the accumulation of both catechin and epicatechin were well correlated with the expression of *TcLAR* and *TcANR*. In exocarp, where both *TcLAR* and *TcANR* had the highest expression among all tissues tested, both catechin and epicatechin were also at maximal levels. In leaves and flowers, where *TcANR* expression was much lower, epicatechin was found at correspondingly lower levels. Likewise in seeds, which exhibited a much lower expression of *TcLAR,* catechin levels were also much lower.

### Functional analysis of the TcANR gene

**Figure 5:**
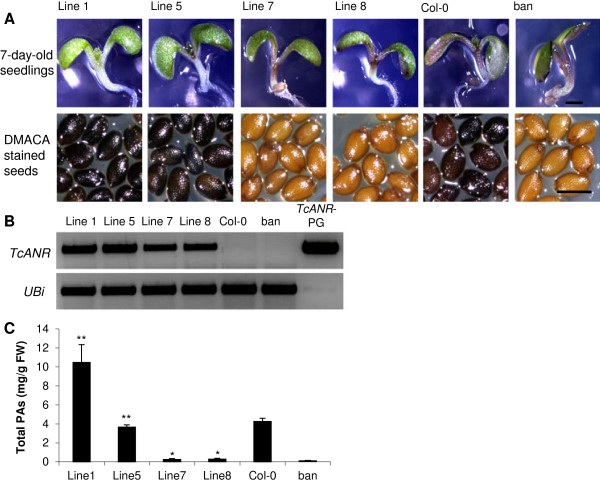
**Complementation of the PA deficient Arabidopsis *ban *mutant phenotype by constitutive expression of *TcANR*. A**, 7-day-old seedlings and DMACA-stained seeds from Col-0 the *ban* mutant (SALK_040250) and four independent transgenic lines (*ban*35S:TcANR). The bar represents 1 mm. **B**, Analysis of *TcANR* and *AtUbi* transcripts in total RNA from leaves of plants shown in **(A)** by semi-quantitative RT-PCR. PCR products from *TcANR*-pGEM plasmid were loaded on the last lane acting as a positive control for the *TcANR* primer set and a negative control for the *AtUbi* primer set. **C**, PA levels in mature seeds of plants shown in **(A)**. PA levels were determined by extraction and DMACA reaction with procyanidin B2 as standards. All data are presented as mean values ± SE, n = 3. *P < 0.05 versus *ban*; **P < 0.01 versus *ban*.

While the sequences and expression patterns of the candidate cacao PA biosynthesis genes were consistent with their candidate functions relative to enzymes from other species, we conducted *in vivo* functional analysis of each gene in transgenic plants to gain direct evidence for their functions. To investigate the *in vivo* function of *TcANR*, a genetic complementation experiment was performed by transferring the *TcANR* coding sequence under the control of an enhanced expression promoter (E12Ω, a modified CaMV35S promoter having the omega arrangement derived from tobacco mosaic virus) into the Arabidopsis *banyuls (ban)* mutant, which is defective in the gene encoding ANR [22]. *ban* mutant seeds are pale yellow due to a lack of PAs, and there is more purple anthocyanin pigment deposited in hypocotyls compared to Arabidopsis (ecotype Columbia) control plants due to increased metabolic flux into this branch of the pathway. From eight independent transgenic *TcANR*-overexpressing lines tested, five lines showed white hypocotyls, and 3 lines showed reduced pigmentation in hypocotyls as compared to *banyuls* mutants. The lines with white hypocotyls also produced seeds exhibiting the wild-type phenotype that stained blue with DMACA reagent, suggesting the deposition of PAs in the seed coat (Figure 5A). After PA extraction and quantification, all lines showed significantly increased levels of PAs (Figure 5C). RT-PCR analysis confirmed expression of *TcANR*, and the expression levels positively correlated with PA accumulation (Figure 5B).

### Functional analysis of TcANS gene

To investigate the *in vivo* function of *TcANS*, two model plants, tobacco and Arabidopsis were utilized. We used an Arabidopsis *ans* (*ldox*) mutant to perform tests of transgenic complementation. We used tobacco as a model system for metabolic flux analysis of the equilibrium between PA and anthocyanin synthesis. Introduction of functional *ANS* genes into tobacco can result in flower petal color changes reflecting alterations in ANS activity.

**Figure 6:**
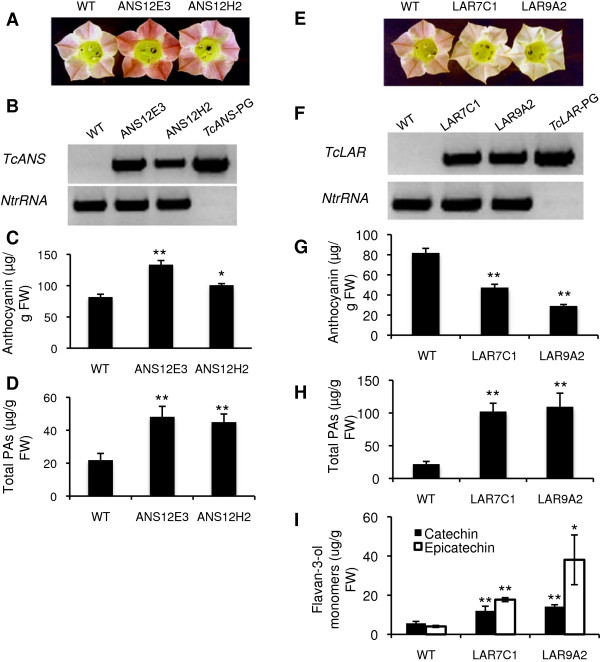
**Characterization of transgenic tobacco flowers constitutively expressing *TcANS *or *TcLAR*. A**, Pigmentation of flower petals from wild type (WT) and two independent lines of *TcANS* transgenic (ANS12E3, ANS12H2) tobacco plants. **B**, Analysis of *TcANS* and *NtrRNA* transcripts in total RNA from leaves of plants shown in **(A)** by RT-PCR. PCR products from TcANS-pGEM plasmid were loaded on the last lane acting as a positive control for *TcANS* primer set and a negative control for the *NtrRNA* primer set. **C**, Anthocyanin levels in flower petals of plants shown in **(A)**. **D**, Total soluble PA levels in flower petals of plants shown in (A). **E**, Flowers from wild type (WT) and two independent lines of *TcLAR* transgenic (LAR7C1, LAR9A2) tobacco plants. **F**, Analysis of *TcLAR* and *NtrRNA* transcripts in total RNA from young leaves of plants shown in (E) by RT-PCR. PCR products from the TcLAR-PGEM plasmid alone were loaded on the last lanes to act as a positive control for the *TcLAR* primer set and a negative control for the *NtrRNA* primer set. **G**, Anthocyanin levels in flower petals of WT and transgenic plants. **H**, Total soluble PAs levels in flower petals of WT and transgenic plants. **I**. Flavan-3-ol accumulation and composition in flowers of wild type (Samsun, ss) tobacco plants and two independent lines of 35S:TcLAR transgenic (LAR7C1, LAR9A2) tobacco plants. Anthocyanin levels were determined by extraction and UV absorption with cyanidin 3-glucoside as standards. Total soluble PA levels are determined by extraction and DMACA reaction with procyanidin B2 as standards. Flavan-3-ol levels were determined by extraction, HPLC separation, and quantification. All data are presented as mean values ± ≥SE,7.*Pn < 0.05 versus WT; **P < 0.01 versus WT.

ANS is involved in both PA synthesis and anthocyanin synthesis, as cyanidin, an anthocyanin precursor, can also be reduced to epicatechin by ANR (Figure 1). This was demonstrated in a recent study describing that down regulation of *MtANS* in *Medicago* resulted in decreased levels of both PAs and anthocyanins [24]. Based on these results, we reasoned that if the putative cacao ANS protein is truly a functional ANS, over-expression of *TcANS* in tobacco should result in increased accumulation of both anthocyanins and PAs. The ORF of *TcANS,* driven by the E12Ω promoter, was introduced into wild-type tobacco (cv. Samsun) for constitutive ectopic expression. Twelve independent hygromycin-resistant lines were generated, of which nine showed a visible increase in pink color intensity in flower petals. The two lines displaying the greatest increase in petal color were chosen for further analysis. RT-PCR analysis confirmed high *TcANS* transcript levels in these two tobacco transgenic lines, which positively correlated with the color of the petals (Figure 6A and B). Amplification of the tobacco ribosomal RNA gene *NtrRNA*, which served as an internal control, showed a relatively similar expression level in both wild-type control and transgenic tobacco plants. As predicted, anthocyanin levels increased in the two transgenic lines (Figure 6C).

The levels of PAs in the petals of transgenic lines, quantified by DMACA assays, were also significantly higher as compared to untransformed Samsun plants (Figure 6D). On average, a two-fold increase of PA was observed in the two lines compared to wild type.

The 35S-*TcANS* transgene was also introduced into the Arabidopsis *ans (ldox)* T-DNA mutant, which produces hypocotyls that appear white to light green due to lack of anthocyanins, and seeds that appear light yellow due to lack of PAs. Eighteen independent hygromycin resistant transgenic T1 seedlings were selected. From these, 2 lines developed wild-type purple colored hypocotyls (Additional file 1: Figure S2) and produced wild-type brown-colored seeds that stained blue after reacting with the DMACA reagent. The color suggested deposition of PAs in the seed coat, however the color intensity was lower than in wild type. RT-PCR using RNA extracted from T2 seedlings confirmed expression of *TcANS* genes (data not shown).

### Functional analysis of the TcLAR gene

Because the *LAR* gene is not present in Arabidopsis [15], we cannot obtain direct evidence of the *in vivo* function of *TcLAR* through genetic complementation analysis in Arabidopsis. Therefore, to functionally characterize *TcLAR*, we used tobacco as a model system. As over-expression of the *ANR* gene can divert the metabolic flow from anthocyanin synthesis to PA synthesis [17], we predicted that over-expression of the cacao *LAR* gene in tobacco would result in a decrease in anthocyanin pigment and an increase in PA accumulation in flower petals. Transgenic tobacco plants were generated that constitutively expressed the ORF of *TcLAR* under the control of the E12Ω promoter. Twenty-two independent transgenic lines that are resistant to hygromycin were generated. Nine of these exhibited a decrease in intensity of the visible pink color of petals. Two lines (Lines LAR7C1 and LAR9A2) exhibited virtually white petals (Figure 6E). RT-PCR showed that both lines expressed high levels of *TcLAR* transcripts (Figure 6F). Quantification of soluble PA and anthocyanin levels indicated that anthocyanin levels in these two lines were about half those of WT controls, and that total PAs accumulated to an approximately 5-fold higher level than in controls (Figure 6G and H).

Moreover, the levels of PAs in transgenic tobacco petals were inversely proportional to the concentrations of anthocyanin, indicating diversion of metabolic flow from anthocyanin to PA synthesis. Insoluble PA levels were also estimated, but the levels were insignificant (data not shown). To confirm the increased accumulation of catechin, as it is the predicted product of TcLAR, we separated and quantified the PA extracts using HPLC. The HPLC analysis showed that there is indeed an approximately 2-fold increase of catechin levels in both transgenic lines. Surprisingly, there is also a significant increase of epicatechin levels (Figure 6I).

**Figure 7:**
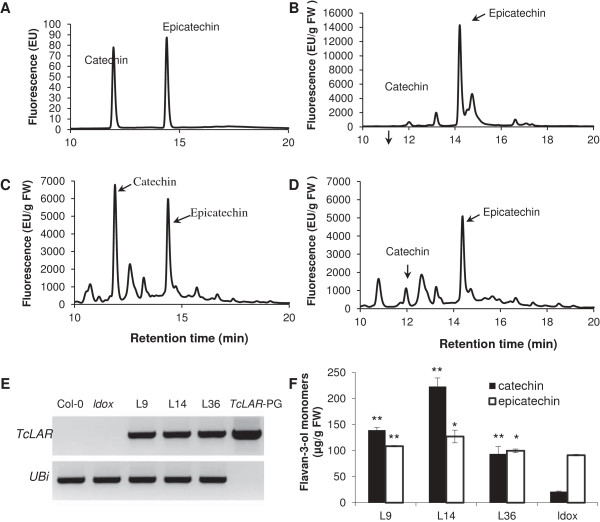
**Complementation of the Arabidopsis PA-deficient *ldox *mutant by constitutively expressing *TcLAR*. A**, Catechin and epicatechin standards analyzed by HPLC. **B**, HPLC analysis of Col-0 young siliques. **C**, HPLC analysis of ldox 35S:TcLAR Line14 young siliques. **D**, HPLC analysis of *ldox* mutant young siliques. **E**. *TcLAR* and *AtUbiquitin* transcripts in total RNA from leaves of ldox 35S:TcLAR transgenic plants, (line 9, line 14 and line 36), Col-O and *ldox* muant plants by RT-PCR. PCR products from the TcLAR-PGEM plasmid were loaded on the last lane as a positive control for the *TcLAR* primer set and as a negative control for the *AtUbiquitin* primer set. **F**, Catechin and epicatechin levels in young siliques of plants that were the source of the total RNA used in **(E)**. Catechin and epicatechin levels were determined by extraction and HPLC analysis. The data are presented as means ± SE, n = 3. *P < 0.05 versus ldox; **P < 0.01 versus ldox. FW, fresh weight; EU, emission units.

We also took advantage of the Arabidopsis *ans (ldox)* T-DNA mutant to examine *LAR* function. Because Arabidopsis lacks an *LAR* gene [12,14,15] and the *ldox* mutant is deficient in cyanidin (the substrate for *ANR*), the *ldox* mutant exhibits a significant decrease of epicatechin and PA synthesis. We reasoned that since the *ldox* mutant accumulates leucoanthocyanidin which can provide the substrate for a heterologous LAR protein, the *ldox* mutant was potentially a good *in vivo* model to analyze LAR function. We predicted that over-expression of *TcLAR* should result in the synthesis of catechin in developing siliques of the *ldox* mutant Arabidopsis, even if PAs were not produced due to lack of epicatechin synthesis. HPLC separation and quantification of PA extracts from Arabidopsis transgenic lines over-expressing *TcLAR* gene in the *ldox* mutant background revealed not only a significant increase of catechin, but also surprisingly, a modest increase of epicatechin (Figure 7). Quantification of total PAs extracted from mature seeds also revealed significant PAs increases in all transgenic lines compared to the *ldox* mutant (Additional file 1: Figure S4).

**Figure 8:**
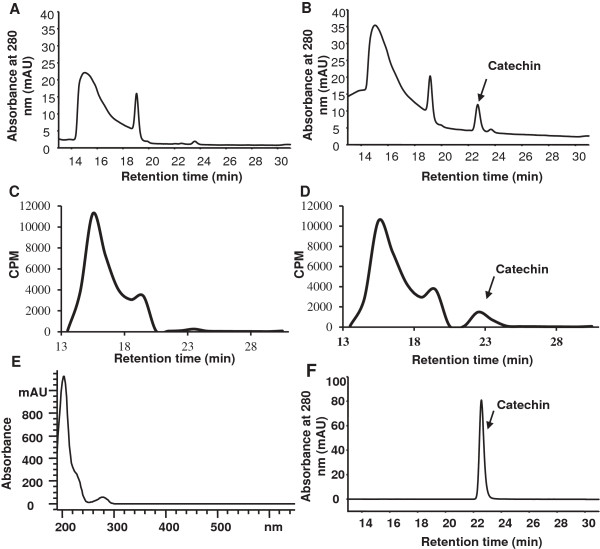
**Enzymatic assay of recombinant TcLAR. A**, HPLC analysis of products from incubation of leucocyanidin with boiled protein as control, monitored by UV spectroscopy. **B**, as above, for incubation of purified recombinant TcLAR with ^3^H-leucocyanidin. **C**, As in **(A)**, but monitoring radioactivity of fractions; **D**, As in **(B)**, but monitoring radioactivity of fractions; (**E**), UV spectrum of catechin product from **(B)**; **F**, HPLC analysis of an authentic standard of (−)-catechin. mAU, milliabsorbance units; CPM, counts per minute.

These unexpected results prompted us to check the possibility that TcLAR may carry a dual enzymatic activity, capable of converting leucoanthocyanidin to both catechin and epicatechin. Thus, recombinant TcLAR protein was expressed in *E. coli*, purified and assayed using ^3^H -labeled leucocyanidin as substrate in the presence of NADPH, followed by analysis of products by HPLC-UV and radioactivity detection. The negative control reaction (boiled protein) showed no product formation (Figure 8A and C), whereas a peak with the same retention time and UV-spectrum as that of the pure (−)-catechin standard (Figure 8E and F) was detected when TcLAR protein was incubated with ^3^H -labeled leucocyanidin (Figure 8B and D). However, no epicatechin product was detected (the retention time of 26–27 min), suggesting that TcLAR does not exhibit duel functionality in this *in vitro* assay.

## Discussion

### The distribution of cacao PA accumulation correlates with expression of the PA biosynthesis genes

The localization of PAs in plant tissues has been well studied in the model plants Arabidopsis, the legume *Medicago truncatula*, and in the fruit species grape (*Vitis vinifera*). In Arabidopsis, the expression of the *AtANR* (*BANYULS*) gene and PA accumulation is limited to the seed coat [22,35]. Similarly in *Medicago* the major localization of PAs is also in the seed coat with very small amounts present in flowers, leaves, roots and stems [24]. Although *Theobroma cacao* and Arabidopsis are phylogenetically closely related, the PA accumulation profiles are quite different. Our results demonstrated that in cacao leaves, flowers and fruits, PAs accumulate at high levels and this is correlated with expression of genes encoding the key enzymes involved in PA synthesis. This is similar to *Vitis*, in which both PA accumulation and genes involved in PA synthesis are found in leaves, fruit skins and seeds [25].

In cacao, the expression of *ANS*, *ANR*, and *LAR* were co-regulated and their expression correlated well with PA accumulation in most of the tissues, suggesting significant roles in PA synthesis for both ANR and LAR. The only exception is in seeds, in which the expression level of *TcANS* is higher than *TcANR* and *TcLAR* is much lower. The high expression level of *TcANS* may contribute to anthocyanin synthesis, because at the time when seed tissues were harvested (18 WAP), anthocyanins are actively synthesized [36-38]. The PA level in seeds is also much lower than in other tissues, well correlated with the *TcLAR* but not *TcANR.* The LAR enzyme seems to be a rate-limiting factor of the PA synthesis. To further explore the individual contributions of these proteins to catechin and epicatechin synthesis, flavan-3-ol monomer composition was examined by HPLC analysis. Cacao flavan-3-ol monomers were composed almost entirely of epicatechin units with catechin units comprising less than 2% in all tissues examined. This result is consistent with aprevious study of PA synthesis in cacao beans [30], in which PA extension units were reported to be composed exclusively of epicatechin, and terminal units were mostly epicatechin with about 1% catechin. Similarly, *Medicago* PAs consist almost entirely of epicatechin units [24]. In grape, both ANR (encoded by *VvANR*) and LARs (encoded by*VvLAR1* and *VvLAR2*) contribute to PA synthesis in the fruits, in which PAs consist of both catechin and epicatechin. However, in grape leaves, although catechin is still present, *LAR* genes are expressed at very low levels.

### Functional characterization of cacao PA biosynthetic genes ANS, ANR and LAR

*In vivo* genetic analysis of *TcANS* and *TcANR* verified their roles in PA biosynthesis. Over-expression of *TcANS* in tobacco resulted in elevated levels of both anthocyanins and PAs in flower petals. It also restored anthocyanin synthesis in hypocotyls as well as PA accumulation in seeds of the Arabidopsis *ans* (*ldox*) mutant. Similarly, over-expression of *TcANR* in the Arabidopsis *banyuls* (*anr*) mutant restored PA synthesis in seeds. Moreover, ectopic over-expression of *TcANR* in hypocotyls diverted the anthocyanin synthesis branch and resulted in decreased anthocyanin pigments related to gene expression levels. The catalytic activities of LAR proteins from grapevine, *Medicago* and *Desmodium* have been verified by *in vitro* assays of recombinant proteins [14,24,25]. *Lotus* LAR showed weak activity in a recombinant DFR protein coupled assay [26]. However, none of the enzyme assays was able to recover sufficient flavan-3-ol products for stereo chemical analysis to allow specific determination of catechin and epicatechin. The *in vivo* activities of *Medicago* and *Desmodium* LAR proteins was also tested in tobacco and white clover [14,24]. In the former study [14], the authors found that transgenic plants expressing the DuLAR protein showed slightly elevated LAR enzymatic activity compared to extracts from untransformed plants, but they did not demonstrate changes in metabolite synthesis. In a more recent study of *MtLAR* overexpresing transgenic tobacco plants, there were no detectable changes in anthocyanin and PA levels [24]. The results presented in this work shows that a cacao *TcLAR* gene is co-regulated with *TcANR* in all tissues examined, providing a simple model to investigate LAR enzyme functions. In our results, *TcLAR* showed clear *in vivo* activities in both transgenic tobacco and Arabidopsis plants by converting metabolic flow from anthocyanin synthesis to PA synthesis. This data provides a direct genetic evidence for a clear role of *LAR* in PA biosynthesis. The metabolic flow divergence resulting from *TcLAR* over-expression could be due to perturbations of a hypothesized metabolic channeling mechanism that suggests that multiple enzymes in each subsequent step of the synthesis pathway interact as a means to increase the efficiency and throughput of the pathway [39].

To our surprise, ectopic expression of *TcLAR* resulted in elevated levels of both catechin and epicatechin units in tobacco flowers (Figure 6H) in contrast to the prediction that only catechin should be formed. Based on the sequence similarity between LAR and ANR, it is possible that LAR could function redundantly to ANR and convert cyanidin to epicatechin. Alternatively, LAR may perform as a dual functional enzyme and convert leucoanthocyanidin to both catechin and epicatechin. To test the possibility that LAR performs the same activity as ANR and uses cyanidin as a substrate to form epicatechin, we took advantage of the Arabidopsis *ldox* (*ans*) mutant. Since Arabidopsis does not have an *LAR* gene and synthesizes only epicatechin through ANS and ANR pathway, the *ldox* mutants have a significantly reduced PA (epicatechin) level due to lack of a supply of cyanidin, the epicatechin precursor. Thus, the *ldox* mutant provides a system that has leucoanthocyanidin but not cyanidin. Over-expression of *TcLAR* in the *ldox* mutant resulted in synthesis of catechin confirming its predicted enzymatic function. However, HPLC quantification showed that there was only a slight elevation of epicatechin levels compared to the significant elevation of catechin, suggesting that LAR was less likely to perform dual function and convert leucoanthocyanidin to epicatechin.

When recombinant TcLAR was assayed *in vitro*, while we observed the production of catechin from 2,3-*trans-*3,4-*cis-*leucoanthocyanidin, we did not detect even trace amounts of epicatechin. The failure to detect epicatechin in assays with the cacao LAR enzyme suggests that it functions solely in converting leucoanthocyanidin to catechin. However, it is possible that LAR enzyme can synthesize epicatechin from 2,3-*cis-*3,4-*trans-*leucocyanidin as suggested by [14]; but we could not test this possibility in the recombinant TcLAR enzyme assay because 2,3-*cis-*3,4-*trans-*leucocyanidin is not commercially available. The increase in epicatechin in *TcLAR* over-expressing Arabidopsis and tobacco plants could be due to the possible existence of a gene(s) encoding a catechin-epicatechin epimerase. One possible candidate gene that could be involved is ANR. *Vitis vinifera* ANR protein expressed and purified from *E.coli* showed epimerase activities *in vitro* and converted cyanidin to a mixture of catechin and epicatechin [40]. Similarly, two recombinant ANR proteins cloned from *Camellia sinensis* also showed epimerase activities *in vitro*[41]. However, we regard this as a remote possibility because Arabidopsis itself does not synthesize catechin and thus it would be surprising if a catechin-epicatechin-specific epimerase were expressed in the absence of its substrate. Nevertheless, we cannot exclude the possible existence of such an epimerase that may also serve other functions important in plant development. A second alternative hypothesis to explain epicatechin formation would be racemization of catechins by polymerization to proanthocyanidins followed by nonstereo-specific depolymerization. An earlier report lends some support to this idea through the analysis of transgenic apple lines in which the *MdANS* gene was silenced. The transgenic lines showed a drastic reduction in anthocyanins together with significant increase of both catechin and epicatechin, concomitant with an increase of catechin derived PA polymers and a decrease in epicatechin derived polymers [42]. Based on these observations, the authors of this study suggested that the increased epicatechins were derived from non-stereospecific depolymerization of proanthocyanidins.

A third possible explanation for our results is the hypothesis that increased production of catechin in the transgenic plants alters the metabolic equilibrium in such a way as to increase flux through the epicatechin branch as a feedback mechanism to balance the relative amounts of catechin and epicatechin. It is known that DuLAR activity can be inhibited by catechin with an IC50 as low as 12 μM [14]. Feedback and feedforward mechanisms also exist at a transcriptional level in the flavonoid pathway. For example, a bean chalcone synthase (CHS) promoter can be stimulated by low concentrations of trans-cinnamic acid (CA), the first intermediate of the phenylpropanoid pathway while its activity can be repressed by high concentrations of CA [43]. On the other hand, high concentrations of trans-p-coumaric acid, the second intermediate of the phenylpropanoid pathway, can still stimulate the CHS promoter [43]. It seem likely that a complex web of homeostatic mechanisms function to control the flux through this entire pathway and elucidation of these mechanisms remains a major objective of this field for the future.

## Conclusions

We successfully isolated three genes encoding key PA synthesis enzymes in *Theobroma cacao*, *TcANS*, *TcANR*, and *TcLAR. In vivo* genetic analysis of *TcANS* and *TcANR* in tobacco and complementation in Arabidopsis verified their roles in PA biosynthesis. *In vitro* enzyme assays of TcLAR recombinant protein verified its predicted function. Moreover, *in vivo* overexpressing *TcLAR* in tobacco and Arabidopsis *ldox* mutants successfully diverted metabolic flow to PA syntheis, which provide direct evidence for a clear role of the *TcLAR* gene in PA biosynthesis. Our results provide new knowledge and genetic tools for development of cacao varieties with novel PA profiles through conventional breeding or genetic approaches.

## Methods

### Plant material

Two *Theobroma cacao* varieties: Scavina 6 and Amelonado were used for this study. Cacao plants were grown in greenhouse as previously described [44]. Leaf and flower tissues were collected from Scavina 6 plants. For leave tissues, young red stage leaves were collected. The definition of leaves stages were previously described [45]. Cacao pods were obtained by hand pollinating Amelonado (a self-compatible variety), in order to reduce the effect of genetic variation on seed traits. Pods harvested 18 weeks after pollination were dissected into exocarp (outer fruit tissue) and seeds for separate analysis. Exocarp samples represent the outer 1–3 mm layer of the fruit obtained using a fruit peeler. All samples were frozen in liquid nitrogen upon collection and stored at −80°C until extraction.

Transgenic and wild-type tobacco plants (*Nicotiana tabacum* var. Samsun provided by Wayne Curtis, Department of Chemical Engineering, The Pennsylvania State University) were grown in a greenhouse under the same condition as cacao plants. Arabidopsis plants (*Arabidopsis thaliana*) were grown in soil at 22°C, 50% humidity and a 16 h/8 h light/dark photoperiod in a growth chamber (Conviron, Pembina, ND, USA). Plants grown aseptically were plated on MS medium [46] with 2% (w/v) sucrose solidified with 0.6% (w/v) agar. Arabidopsis ecotype Columbia (Col −0) plants were used as the wild type. T-DNA insertion mutants *ban* (SALK_040250) and *ldox* (SALK_028793) were obtained from The Arabidopsis Biological Resource Center (Columbus, OH, USA).

### Nucleic acid purification and cDNA synthesis

Total RNA from leaves of *Theobroma cacao* (Scavina 6) was isolated using a modified cetyl trimethyl ammonium bromide (CTAB) extraction method as previously described [47] with the following modifications. RNA isolated from the CTAB extraction LiCl precipitation was further purified and concentrated using RNeasy columns (Qiagen, Valencia, CA, USA), but the phenol/chloroform extraction and sodium acetate/ehanol precipitation step was omitted. The quality of RNA was verified by observing absorbance ratios of A260/A280 (1.8 to 2.0) and A260/A230 (1.8 to 2.2) and by separating 200 ng RNA samples on 0.8% agarose gels to examine intact ribosomal bands. First strand cDNA was synthesized using the SMART RACE cDNA amplification kit (Clontech, Mountain View, CA, USA).

### Isolation of cDNA and genomic clones from *Theobroma cacao*

The putative expressed sequence tag (EST) sequences of cacao anthocyanidin reductase (*TcANR)*, anthocyanidin synthase (*TcANS)* and leucoanthocyanidin reductase (*TcLAR)* genes were obtained by searching the *Theobroma cacao* EST database (http://esttik.cirad.fr/) [31] using the tBLASTn program [48]. The query sequences used were the protein sequences of BANYULS and LDOX from *Arabidopsis thaliana* and DuLAR from *Desmodium uncinatum* respectively (Accession numbers: NP_176365, Q96323 and CAD79341). Based on the sequences of the EST contigs from the ESTtik database (EST Treatment and Investigation Kit; http://esttik.cirad.fr), PCR primers were designed to amplify the entire coding sequences of each gene: ANR_F (5′-AG*CC*ATGGCCAGCCAGACCGTAGG-3′) and ANR_R (5′-*GCGGCCGC*TCACTTGAGCAGCCCCTTAGC-3′), ANS_F (5′-*CCATGG*TGACTTCAATGGCCCCCAG-3′) and ANS_R (5′-*GCGGCCGCC*TCAATTAGACAGGCCATC-3′) and LAR_F (*CCATGG*ATATGAAATCAACAAACATGAATGGTTC) and LAR_R (*GCGGCCGC*TCATGTGCATATCGCAGTG). *Nco*I sites were added to the 5’ end of each start codon and *Not*I sites were added to the 3’ end of each stop codon to facilitate the subsequent cloning into binary T-DNA vectors. The coding sequences were amplified from cacao cDNA prepared from young leaves (genotype Scavina 6) with the Advantage cDNA PCR Kit (Clontech, Mountain View, CA, USA) using these primers. PCR reactions were carried out in a total volume of 20 μL at 94°C for 5 min; 5 cycles of 94°C for 30 sec, 55°C for 30 sec, and 72°C for 1 min; then another 23 cycles of 94°C for 30 sec, 60°C for 30 sec, and 72°C for 1 min; followed by a final extension at 72°C for 5 min. PCR products were gel purified and cloned into the pGEM-T easy vector (Promega, Madison, WI, USA). The correct open reading frames (ORFs) of each of the resulting constructs (pGEMT-TcANR, pGEMT-TcANS and pGEMT-TcLAR) were confirmed by DNA sequencing.

The DNA sequences of the *TcANR*, *TcANS* and *TcLAR* genes were obtained by isolation and sequencing of genomic clones. Briefly, 2 high-density filters were arrayed with 18,432 colonies of *Theobroma cacao* (genotype LCT-EEN 37) bacterial artificial chromosome (BAC) clones on each (library and filters constructed by The Clemson University Genomics Institute (CUGI, https://www.genome.clemson.edu/). The filters were hybridized to full-length cDNA of each gene labeled with P^32^ using the MEGA Labeling Kit (GE Healthcare, Piscataway NJ). DNA hybridizations were carried out at 65°C in 1 mM ethylenediaminetetraacetic acid (EDTA), 7% sodium dodecyl sulfate (SDS), 0.5 M sodium phosphate (pH7.2) for 16–18 h. Filters were washed twice at 65°C in 1 mM EDTA, 1% SDS, 40 mM Na_2_HPO_4_ for 20 min, twice at 65°C in 1.5× sodium chloride/sodium citrate (SSC), 0.1% SDS for 20 min and twice at 65°C in 0.5× SSC, 0.1% SDS for 20 min. Two or more BAC clones were identified for each gene and confirmed by PCR using plasmid DNA from individual colonies and gene specific primers. High purity plasmid DNA from individual BAC clones was then isolated using a NucleoBond BAC 100 kit (Macherey-Nagel Inc., Bethlehem, PA, USA) and both strands of DNA were sequenced using primers designed from the cDNAs and genomic DNA. DNA sequencing results were analyzed and assembled using Vector NTI software (Invitrogen, San Diego, CA), application Contig Assembly. The DNA sequence of each gene was then compared to the corresponding coding sequence by using the BLAST2 online tool (http://www.ncbi.nlm.nih.gov/blast/bl2seq/wblast2.cgi) to obtain exon and intron locations for the gene organization analyses.

### Phylogenetic analysis

Deduced protein sequences of all Arabidopsis *IFR*-like genes were retrieved from The Arabidopsis Information Resource (TAIR) database (http://www.arabidopsis.org/) by querying the TAIR protein database with the *Desmodium* LAR protein sequence (CAD79341) using the WU-BLAST2 (BLASTP) program. Protein sequences from other species were retrieved from the GenBank (http://www.ncbi.nlm.nih.gov/Genbank/). Accession numbers are indicated in the figure legend.

Multiple sequence alignment of proteins was performed by ClustalX algorithm [49] with default parameter settings (gap opening penalty: 10, gap extension penalty: 0.2, delay divergent cutoff: 30%, protein weight matrix: Gonnet series) and this alignment was used to construct the phylogenetic tree using the neighbor-joining method in the MEGA package [50]. Five hundred bootstrapped datasets were used to estimate the confidence of each tree clade.

### Determination of proanthocyanidins (PAs) and anthocyanins

For quantitative analysis of anthocyanin levels in transgenic tobacco flowers, fresh petals (0.3-0.5 g fresh weight) from three flowers were immersed in 5 mL ethanol: 6 M HCl (1:1) and incubated at 4°C overnight. The extract solution was transferred to a new tube and the petals were extracted for the second time using the same method. Absorbance of the pooled extract solution was then measured at 526 nm and the total anthocyanin levels were calculated using a standard molar absorbance curve prepared using cyanidin-3-glucoside (Sigma-Aldrich, MO, USA).

To extract soluble PAs from cacao and tobacco tissues, 0.3 to 0.5 g of frozen tissues were ground into a fine powder in liquid nitrogen and then extracted with 5 mL of extraction solution (70% acetone: 29.5% water: 0.5% acetic acid) by vortexing for 5 seconds followed by water bath sonication for 15 min using a bench top ultrasonic cleaner (Model 2510, Bransonic, Danbury, CT, USA). To extract soluble PAs from Arabidopsis seeds and siliques, the same extraction solution and method were applied, except that 100 to 500 mg dry seeds and 10 green siliques were used as grinding samples, and 500 μL extraction solution were used. After sonication, samples were vortexed again and centrifuged at 2500 g for 10 min. The supernatant was transferred to a new tube and the pellet was re-extracted twice as above. Pooled supernatants were extracted twice with hexane to remove fat and chlorophyll and then filtered through a 0.45 μm polytetrafluoroethylene (PTFE) syringe filter (Millipore, Billerica, MA, USA). Depending on availability of plant samples, different numbers of biological replicates were performed for cacao, tobacco and Arabidopsis samples. For cacao, there are at least five biological replicates, for tobacco, there are seven or more biological replicates, and for Arabidopsis, there are three biological replicates.

To quantify soluble PA levels, extracts were then quantified by reaction with *p-*dimethylamino-cinnamaldehyde (DMACA), which specifically interacts with PA monomers and polymers to form blue pigments [51]. Briefly, 50 μL aliquots of samples were mixed with 200 μL of dimethylaminocinnamaldehyde (DMACA; Sigma-Aldrich, MO, USA) reagent (0.1% DMACA, 90% reagent-grade ethanol, 10% HCl) in 96-well microtiter plates. Absorption was measured at 640 nm at one-minute intervals for 20 min to get the highest readings. Triple technical replicates were performed to obtain mean values. The total PA levels were calculated using the standard molar absorbance curve prepared using procyanidin B2 (Indofine, NJ, USA).

For quantitative analysis of insoluble PAs from cacao tissues, the residues from soluble PA extractions were air dried in an exhaust hood for two days, weighed, and 5 mL butanol-HCl reagent (95% butan-1-ol: 5% concentrated HCl) was added and the mixture was sonicated for one hour followed by centrifugation at 2500 g for 10 min. An aliquot of clear supernatant was diluted 40-fold in butanol-HCl reagent and absorbance was measured at 550 nm to determine the amount of background absorption. The samples were then boiled for 1 hour with vortexing every 20 min, cooled to room temperature and centrifuged again at 2500 g for 10 min. The supernatant from boiled sample was diluted 40-fold in butanol-HCl reagent and absorbance was measured at 550 nm. The values were normalized by subtraction of the background absorbance and the PA levels were calculated as cyanidin equivalents using cyanidin-3-glucoside (Sigma-Aldrich, MO, USA) as standards.

To visualize the presence of PAs in Arabidopsis seeds, dry seeds were immersed for 2 days in the 0.1% DMACA reagent described above and then washed 3 times with 70% ethanol as described previously [35]. Catechin and epicatechin content was determined by reverse-phase HPLC using an Alliance separations module (Model 2695; Waters, Milford, MA, USA) equipped with a multi λ fluorescence detector (Model 2475; Waters, Milford, MA, USA). Samples of soluble PA extracts (10 μL) were separated on a 250 mm × 4.6 mm Luna 5-μm Phenyl Hexyl column (Phenomenex, Torrance, CA, USA) and then assayed by fluorescence emission at 315 nm following excitation at 280 nm. The HPLC separation utilized a binary mobile phase gradient mixture of A+B where mobile phase A was 0.5% trifluoroacetic acid (TFA) (v/v with water) and mobile phase B was 0.5% TFA (v/v with methanol). The gradient conditions were: 0 min, 16% mobile phase B; 4 min, 16% mobile phase B; 14 min, 50% mobile phase B; 18 min, 50% mobile phase B; 22min, 100% mobile phase B; 26 min, 100% mobile phase B; 30 min, 16% mobile phase B. The column was maintained at 30°C and the flow rate was 1 mL/min. Catechin and epicatechin standards were purchased from Sigma-Aldrich (St. Louis, MO, USA). This work was performed at the Hershey Technical Center (Hershey, PA, USA).

### Transformation of tobacco and arabidopsis

The coding sequences of *TcANS, TcANR* and *TcLAR* were excised from the cloning vector (pGEM-T easy) (Promega, Madison, WI, USA) with *Nco*I and *Not*I restriction enzymes and cloned into the pE2113-EGFP [44] intermediate vector to replace the original EGFP coding sequence. As a result, the cacao coding sequences are located immediately downstream of the E12-Ω, an enhanced expression promoter modified from CaMV35S [52], and upstream of the CaMV35S-terminator. The over-expression cassettes of *TcANR* and *TcANS* was excised out from pE2113-TcANR and pE2113-TcANS constructs respectively with *Hae*II restriction enzyme, blunt ended with T4 polymerase and then introduced into the pCAMBIA-1300 binary vector (CAMBIA, Canberra, Australia) linearized with *Sma*I restriction enzyme; the over-expression cassettes of *TcLAR* was excised out from pE2113-TcLAR construct with *Hind*III and *Pvu*II restrictin enzyme and ligated into pCAMBIA-1300 binary vector linearized with *Hind*III and *Sma*I restriction enzyme. All binary transformation constructs were introduced into *Agrobacterium tumefaciens* strain AGL1 [53] by electroporation as described previously [54].

Tobacco leaf disc transformation was performed as previously described [55] and transgenic shoots were regenerated on MSs (MS shooting) media supplemented with 25 mg/L hygromycin. Only one shoot was selected from each explant to ensure independent transformants. After rooting for 2 weeks in MSr (MS rooting) media supplemented with 25 mg/L hygromycin, hygromycin-resistant plantlets were transferred to soil and grown in a greenhouse as described above.

Arabidopsis transformation was carried out using the floral dip method [56], and T1 transgenic plants were selected on MS media supplemented with 2% sucrose, 0.65% agar and 25 mg/L hygromycin. Hygromycin-resistant T1 seedlings were transferred to soil 7 days after germination and grown in a growth chamber as described in above.

### Expression analysis of TcANS, TcANR and TcLAR

Total RNA from leaves, flowers, whole pods, pod exocarp and ovules of *Theobroma cacao* (Scavina 6 and Amelonado) was isolated as described above. Total RNA from young leaves of transgenic and wild-type tobacco plants as well as Arabidopsis plants was isolated using the RNeasy Plant mini kit (Qiagen, Valencia, CA, USA). cDNA was synthesized from 1 μg of total RNA in a total volume of 20 μL using M-MuLV Reverse Transcriptase (NEB, Ipswich, MA, USA) according to the supplier’s protocols, and 2 μL were used in the subsequent reverse transcription-PCR (RT-PCR) reactions. The primers for RT-PCR were designed to amplify across at least one intron giving products of approximately 500 bp from cDNA and 700 bp to 1500 bp from genomic DNA. These primer sets were used to check all cDNAs for genomic DNA contamination. The primers used for *TcANS* were TcANSRT_F (5′-ACCTTGTTAACCATGGGATCTCGG-3′) and TcANSRT_R (5′-GACGGTGTCACCAATGTGCATGAT-3′); the primers used for *TcANR* were TcANR_F (5′- TGCTTGAGAAGGGCTACGCTGTTA-3′) and TcANR_R (5′-AAAGATGTGGCAAGGCCAATGCTG-3′); the primers used for *TcLAR* were TcLAR_F (5′-AATTCCATTGCAGCTTGGCCCTAC-3′) and TcLAR_R (5′-GGCTTGCTCACTGCTTTGGCATTA-3′). *TcActin* was used as an internal standard for cacao gene expression using primer set Tc46RT_F (5′-AGCTGAGAGATTCCGTTGTCCAGA-3′) and Tc46RT_R (5′-CCCACATCAACCAGACTTTGAGTTC-3′). *AtUbi* and *NtrRNA* was chosen as constitutive expression controls for Arabidopsis (ubiquitin) and tobacco (rRNA) respectively with primer pairs AtUbi_F (5′-ACCGGCAAGACCATCACTCT-3′) and AtUbi_R (5′-AGGCCTCAACTGGTTGCTGT-3′) [57], and NtrRNA_F (5′-AGGAATTGACGGAAGGGCA-3′) and NtrRNA_R (5′-GTGCGGCCCAGAACATCTAAG-3′) [58].

The number of PCR cycles was optimized between 20 and 32 to select a cycle number such that amplification was in the linear range; 28 cycles were chosen for all the RT-PCR reactions. The PCR reaction was carried out in a total volume of 20 μL at 94°C for 5 min; 28 cycles of 94°C for 30 sec, 55°C for 30 sec, and 72°C for 45 sec; followed by a final extension at 72°C for 5 min. The PCR products were visualized on 1% agarose gels stained with ethidium bromide (EtBr) and documented using Molecular Imager Gel Doc XR + System equipped with a 16-bit CCD camera (Bio-Rad Laboratories, Hercules, CA) and bands were quantified using Quantity One 1-D Analysis Software (Bio-Rad Laboratories, Hercules, CA).

### Assay of LAR activities

The open reading frame (ORF) of the TcLAR gene was PCR amplified from pGEMT-TcLAR using Advantage 2 polymerase mix (Clontech, Mountain View, CA) and the following primers: TcLARCDF1 (5′-*GAGCTC***atg**gatatgaaatcaacaaacatg-3′; the *Sac*I site is in italics and the start codon is bold) and TcLARCDR2 (5′-24 *CTCGAG*tgtgcatatcgcagtg-3′; the *Xho*I site is in italics and the stop codon was removed to incorporate the C-terminal His-tag sequence of the expression vector at the 3’ end of the ORF of TcLAR). It was then subcloned into the *Sac*I and *Xho*I sites of the pET-21a expression vector (Novagen, Gibbstown, NJ, USA). After confirmation by sequencing, the resulting vector pET21a-TcLAR was transformed into *Escherichia coli* strain Rosetta (DE3) (Novagen, Gibbstown, NJ, USA). For protein expression, a single bacterial colony was inoculated into Luria-Bertani medium (10 g/L tryptone, 5 g/L yeast extract, 10 g/L NaCl) containing 100 μg/mL ampicillin and grown at 37°C overnight. An overnight culture was then diluted into terrific broth (TB) medium (12 g/L Tryptone, 24 g/L Yeast Extract, 0.4% glycerol, 2.31 g/L KH2PO4, 12.54 μg/mLg/L K2HPO4) containing 100 ampicillin and grown at 37°C until the OD600 reached 0.6-0.8, at which time IPTG (isopropyl β -D-1-thiogalactopyranoside), was added to a final concentration of 1 mM to induce protein expression. Recombinant TcLAR protein with a 6⋅His tag at the C terminus was purified using a Magne-His kit (Promega, Madison, WI, USA) and the protein concentration measured by the Bradford method [59]. This work was performed at the Samuel Roberts Nobel Foundation (Ardmore, Oklahoma, USA).

^3^H-3,4-cis-leucocyanidin was synthesized as described by [60]. Assay of recombinant TcLAR protein with ^3^H-3, 4-cis-leucocyanidin was carried out in a final volume of 100 μL containing 10% (w/v) glycerol, 100 mM potassium phosphate (pH 7.0), 4 mM dithiothreitol (DTT), 0.5 mM NADPH, 0.4 mM ^3^H-leucocyanidinμgofpurifieand recombinant30 TcLAR protein. The reaction was initiated by the addition of enzyme and incubated at 30°C for 1 h. The assay was terminated by the addition of 20 μL of methanol followed by centrifugation. Products were analyzed by HPLC, with absorbance monitoring at 280 nm. Products eluting at retention times between 13 to 31 min were collected (1 min/tube) and the fractions containing labeled products were identified by liquid scintillation counting. Boiled pure protein was used as a control.

Reverse-phase HPLC analysis of enzymatic products was performed using an Agilent HP1100 HPLC (Agilent Technologies, Inc., Santa Clara, CA, USA) with the following gradient using solvents A (1% phosphoric acid) and B (acetonitrile) at a 1 mL/min flow rate: 0 to 5 min, 6% B; 5 to 10 min, 6% to 10% B; 10 to 20 min, 10% to 11% B; 20 to 25 min, 11% to 12.5% B; 25 to 45 min, 12.5% to 37% B; 45 to 48 min, 37% to 100% B; 48 to 58 min, 100%, 58 to 60 min 100% to 6% B. Absorbance data were collected at 280 nm. Identifications were based on comparison of chromatographic behavior and UV spectra with authentic standards. This work was performed at Samuel Roberts Nobel Foundation (Ardmore, Oklahoma, USA).

Sequence data from this article can be found in the GenBank/EMBL data libraries under accession numbers GU324347 (*TcANR*), GU324349 (*TcANS*) and GU324351 (*TcLAR*).

## Competing interests

The authors declare that they have no competing interests.

## Authors’ contributions

YL performed most of the experiments, ie, sequence analysis, gene cloning, gene expression studies, transgenic tobacco and Arabidopsis generation, phenotypic analysis of transgenic lines, and drafted the manuscript. ZS participated in gene expression analysis, transgenic Arabidopsis lines generation and analysis. SNM participated in the design of the study, directed the vector construction and tobacco transgenic lines generation, and participated in drafting of the manuscript. MJP developed the catechin and epicatechin HPLC quantification assays and directed PA quantification analysis. MJG conceived the study, drafted the manuscript and gave advice on experimental design, data analysis and execution. All authors read and approved the final manuscript.

## Supplementary Material

Additional file 1 **Figure S1.** Multiple sequence alignment of the LAR, ANS and ANR proteins as well as related IFR and DFR proteins of the RED superfamily. **Figure S2**. Complementation of the PA and anthocyanin deficient *ans* (*ldox)* mutant phenotype by constitutively expressing *TcANS*. **Figure S3**. HPLC analysis of extracts from transgenic Arabidopsis and tobacco key samples with and without standard spikes to validate the peak of catechin and epicatechin. **Figure S4**. Complementation of the PA deficient Arabidopsis ldox mutant phenotype by constitutive expression of TcLAR. Click here for file
