# Supplementary material for: Proanthocyanidin synthesis in Theobroma cacao: genes encoding anthocyanidin synthase, anthocyanidin reductase, and leucoanthocyanidin reductase
Source: BMC Plant Biol. 2013 Dec 5;13:202. doi: 10.1186/1471-2229-13-202 (PMC4233638; doi:10.1186/1471-2229-13-202)
Supplement: Additional file 1 — Figure S1. Multiple sequence alignment of the LAR, ANS and ANR proteins as well as related IFR and DFR proteins of the RED superfamily. Figure S2. Complementation of the PA and anthocyanin deficient ans (ldox) mutant phenotype by constitutively expressing TcANS. Figure S3. HPLC analysis of extracts from transgenic Arabidopsis and tobacco key samples with and without standard spikes to validate the peak of catechin and epicatechin. Figure S4. Complementation of the PA deficient Arabidopsis ldox mutant phenotype by constitutive expression of TcLAR. [file 1471-2229-13-202-S1.pdf]

```
                         *        20         *        40         *        60
AT1G75290 : ----------MASE----KSKILVIGGTGHIGKLIIEASVKAGHSTLALVREASLSDPNK :  46
AT1G75280 : ----------MATE----KSKILVIGGTGYIGKFLVEASAKAGHSTFALVREATLSDPVK :  46
AT1G75300 : ----------MTTE----KSKILVIGGTGYMGEFIVEGSAKAGNPTFALVREASLSDPVK :  46
AT1G19540 : ----------MT-------SKILVIGATGLIGKVLVEESAKSGHATFALVREASLSDPVK :  43
AT4G39230 : ----------MTS-----KSKILFIGGTGYIGKYIVEASARSGHPTLVLVRNSTLTSPSR :  45
AT4G34540 : ----------MEEEK--KKSRVLIIGATGRLGNYLTRFSIESGHPTFALIRNTTLSDKLK :  48
AT4G13660 : ----------MKETNFGEKTRVLVVGGTGSLGRRIVSACLAEGHETYVLQRPEIGVDIEK :  50
AT1G32100 : ----------MGESKRTEKTRVLVVGATGYIGKRIVRACLAEGHETYVLQRPEIGLEIEK :  50
TcLAR     : MDMKSTNMNGSSPNVSEETGRTLVVGSGGFMGRFVTEASLDSGRPTYILARSSSNS-PSK :  59
VvLAR     : -------MTVSP--VPSPKGRVLIAGATGFIGQFVAAASLDAHRPTYILARPGPRS-PSK :  50
LcLAR1-1  : -------MVSTAATPPATAGRILIIGATGFMGQFMTKASLGLGRSTYLLLRPGSLT-PSK :  52
LCLAR1-2  : -------MVSTAATPPATAGRILIIGATGFMGQFMTKASLGLGRSTYLLLRPGSLT-PSK :  52
MtLAR     : ------MAPSSSPTTPISKGRVLIVGATGFMGKFVTEASISTAHPTYLLIRPGPLI-SSK :  53
DuLAR     : -------MTVSGAIPSMTKNRTLVVGGTGFIGQFITKASLGFGYPTFLLVRPGPVS-PSK :  52
MtDFR     : --------------MGSMAETVCVTGASGFIGSWLVMRLMERGYMVRATVRDPENLKKVS :  46
AtDFR     : --------------MVSQKETVCVTGASGFIGSWLVMRLLERGYFVRATVRDPGNLKKVQ :  46
ZmA1      : ---------MERGAGASEKGTVLVTGASGFVGSWLVMKLLQAGYTVRATVRDPANVGKTK :  51
AtBAN     : ---------MDQTLTHTGSKKACVIGGTGNLASILIKHLLQSGYKVNTTVRDPENEKKIA :  51
LcANR     : ---------MASIKPIYEKKKACVIGGTGFVASLLIKQLLEKGYAVNTTVRDPDNHKKIS :  51
TcANR     : ----------MASQTVG-KKTACVVGGTGYVASLLVKLLLEKGYAVNTTVRDPDNQKKIP :  49
VvANR     : ---------MATQHPIG-KKTACVVGGTGFVASLLVKLLLQKGYAVNTTVRDPDNQKKVS :  50
CsANR     : ----------MEAQPTA-PKAACVVGGTGFVAATLIKLLLEKGYAVNTTVRDPGNQKKTS :  49
ZmA2      : -----MESSPLLQLPAARVEALSLSGLSAIPPEYVRPADERAG-LGDAFDLARTHANDHT :  54
PfANS     : -------MVTSAMGPSPRVEELARSGLDTIPKDYVRPEEELKSIIGNILAEEKSSE---- :  49
AtANS     : -------------MVAVERVESLAKSGIISIPKEYIRPKEELES-INDVFLEEKKED---- :  43
MtANS     : -----------MGTVAQRVESLALSGISSIPKEYVRPKEELAN-IGNIFDEEKKE----- :  43
TCANS     : ----------MVTSMAPRVESLASSGIQSIPKEYIRPQEELTS-IGNVFEEEKKEE---- :  45
                                        G  g     6              r

                         *        80         *       100         *       120
AT1G75290 : GKTVQNFKDFGVTLLHGDLNDHESLVKAIK--QADVVISTVG------------SMQILD :  92
AT1G75280 : GKTVQSFKDLGVTILHGDLNDHESLVKAIK--QVDVVISTVG------------SMQILD :  92
AT1G75300 : SKTIQSFKDLGVTILHGDLNDHESLVKAIK--QVDVVISTIG------------HKQIFD :  92
AT1G19540 : AQLVERFKDLGVTILYGSLSDKESLVKAIK--QVDVVISAVGRF----------QTEILN :  91
AT4G39230 : SSTIENFKNLGVQFLLGDLDDHTSLVNSIK--QADVVISTVG------------HSLLGH :  91
AT4G34540 : S-----LSDAGVTLLKGSLEDEGSLAEAVS--KVDVVISAIP------------SKHVLD :  89
AT4G13660 : VQLLLSFKRLGAHLVEGSFSDHQSLVSAVK--QVDVVVSAMSGVHFR-------THNIPV : 101
AT1G32100 : VQLFLSFKKLGARIVEGSFSDHQSLVSAVK--LVDVVVSAMSGVHFR-------SHNILV : 101
TcLAR     : ASTIKFLQDRGATVIYGSITDKEFMEKVLKEHKIEVVISAVG------------GGSILD : 107
VvLAR     : ANIFKALEDKGAIIVYGLINEQEAMEKILKEHEIDIVVSTVG------------GESILD :  98
LcLAR1-1  : AAIVKSFQDRGAKVIHGVINDKELMVKILKDYEIDVVISLVG------------GGNLMD : 100
LCLAR1-2  : AAIVKSFQDRGAKVIHGVINDKELMVKILKDYEIDVVISLVG------------GGNLMD : 100
MtLAR     : AATIKTFQEKGAIVIYGVVNNKEFVEMILKKYEIDTVISAIG------------AESLLD : 101
DuLAR     : AVIIKTFQDKGAKVIYGVINDKECMEKILKEYEIDVVISLVG------------GARLLD : 100
MtDFR     : HLLELPGAKGKLSLWKADLGEEGSFDEAIKGCTGVFHVATPMDFESKDPENEMIKPTIKG : 106
AtDFR     : HLLDLPNAKTLLTLWKADLSEEGSYDDAINGCDGVFHVATPMDFESKDPENEVIKPTVNG : 106
ZmA1      : PLMDLPGATERLSIWKADLAEEGSFHDAIRGCTGVFHVATPMDFLSKDPENEVIKPTVEG : 111
AtBAN     : HLRKLQ-ELGDLKIFKADLTDEDSFESSFSGCEYIFHVATPINFKSEDPEKDMIKPAIQG : 110
LcANR     : HLLVLQ-SLGELNIFGGELTVENDFDTPIEGSELVFQLATPVNFASEDPENDMIKPAIKG : 110
TcANR     : HLVTLQ-KLGDLKIFRADLTDEGSFDVPIAGCDLVFHVATPVNFASQDPENDMIKPAIQG : 108
VvANR     : HLLELQ-ELGDLKIFRADLTDELSFEAPIAGCDFVFHVATPVHFASEDPENDMIKPAIQG : 109
CsANR     : HLLALK-GSGNLKIFRADLTDEQSFDTPVAGCDLVFHVATPVNFASEDPENDMIKPAIQG : 108
ZmA2      : APRIPVVDISPFLDSSSQQQRDECVEAVRAAAADWGVMHIAGHGIPAELMDRLRAAGTA : 114
PfANS     : GPQLPTIDLEEMD--SRDEEGRKKCHEELKKAATDWGVMHLINHGIPEELIDRVKAAGKE : 107
AtANS     : GPQVPTIDLKNIE--SDDEKIRENCIEELKKASLDWGVMHLINHGIPADLMERVKKAGEE : 101
MtANS     : GPQVPTIDLKEIN--SSDEIVRGKCREKLKKAAEEWGVMHLVNHGISDDLINRLKKAGET : 101
TCANS     : GPQVPTIDLKEID--SEDREVRERCRQELKRAATEWGVMHLVNHGISDELMERVKKAGQK : 103
                                                    6
```

```
                     *       140         *       160         *       180
AT1G75290 : QTKIISAIKEAGNVKRFLPSEFGMDVDKS-SAVEPAKSAFGRKLQTRRDIEAEGIPYTYL : 151
AT1G75280 : QTKIISAIKEAGNVKRFLPSEFGVDVDRT-SAVEPAKSAFAGKIQIRRTIEAEGIPYTYA : 151
AT1G75300 : QTKIISAIKEAGNVKRFLPAEFGIDVERT-SAVEPAKSLFAGKVQIRRAIEAEGIPYTYV : 151
AT1G19540 : QTNIIDAIKESGNVKRFLPSEFGNDVDRT-VAIEPTLSEFITKAQIRRAIEAAKIPYTYV : 150
AT4G39230 : QYKIISAIKEAGNVKRFFPSEFGNDVDRV-FTVEPAKSAYATKAKIRRTIEAEGIPYTYV : 150
AT4G34540 : QKLLVRVIKQAGSIKRFIPAEYGANPDKT-QVSDLDHDFYSKKSEIRHMIESEGIPYTYI : 148
AT4G13660 : QLKLVAAIKEAGNVKRFLPSEFGMDPSRMGHAMPPGSETFDQKMEIRNAIKAAGISHTYL : 161
AT1G32100 : QLKLVEAIKEAGNVKRFLPSEFGMDPPRMGHALPPGRETFDQKMEVRQAIEAAGIPYTYV : 161
TcLAR     : QFNLIEAIRNVDTVKRFLPSEFGHDTDRA-DPVEPGLTMYEQKRQIRRQVEKSGIPYTYI : 166
VvLAR     : QIALVKAMKAVGTIKRFLPSEFGHDVNRA-DPVEPGLNMYREKRRVRQLVEESGIPFTYI : 157
LcLAR1-1  : QRTLVDAIKSVKTVKRFLPSEFGHDTDRA-NPVEPGLTMYKEKRLIRRLIEESGIPYTYI : 159
LCLAR1-2  : QRTLVDAIKSVKTVKRFLPSEFGHDTDRA-NPVEPGLTMYKEKRLIRRLIEESGIPYTYI : 159
MtLAR     : QLTLVEAMKSIKTIKRFLPSEFGHDVDRA-DPVEPGLAMYKQKRLVRRVIEESGVPYTYI : 160
DuLAR     : QLTLIEAIKSVKTIKRFLPSEFGHDVDRT-DPVEPGLTMYKEKRLVRRAVEEYGIPFTNI : 159
MtDFR     : VLDIMKACLKAKTVRRFIFTSSAGTLNVTEDQKP--LWDESCWSDVEFCRRVKMTGWMYF : 164
AtDFR     : MLGIMKACVKAKTVRRFVFTSSAGTVNVEEHQKN--VYDENDWSDLEFIMSKKMTGWMYF : 164
ZmA1      : MISIMRACKEAGTVRRIVFTSSAGTVNLEERQRP--VYDEESWTDVDFCRRVKMTGWMYF : 169
AtBAN     : VINVLKSCLKSKSVKRVIYTSSAAAVSINNLSGTGIVMNEENWTDVEFLTEEKPFNWGYP : 170
LcANR     : VLNVLKSCARAK-VKRVILTSSAASVTIGELKGTDLVMDESNWTDVEFLSNAKPPTWGYP : 169
TcANR     : VLNVLKACAKAKTVKRVVLTSSAAAVSINTLKGTDLVLTEKDWTDVEFLSSAKPPTWGYP : 168
VvANR     : VVNVMKACTRAKSVKRVILTSSAAAVTINQLDGTGLVVDEKNWTDIEFLTSAKPPTWGYP : 169
CsANR     : VVNVLKACAKAGTVKRVILTSSAAAVSINKLNGTGLVMDESHWTDTEFLNSAKPPTWGYP : 168
ZmA2      : FFALPVQDKEAYANDPAAGRLQGYGSRLATNTCGQREWEDYLFHLVHPDGLADHALWPAY : 174
PfANS     : FFELPVEEKEAYANDQAAGNVQGYGSKLANNASGQLEWEDYFFHCVYPEHKTDLSIWPTK : 167
AtANS     : FFSLSVEEKEKYANDQATGKIQGYGSKLANNASGQLEWEDYFFHLAYPEEKRDLSIWPKT : 161
MtANS     : FFELPVEEKEKYANDQSSGKIQGYGSKLANNASGQLEWEDYFFHCIFPEDKRDLSIWPKT : 161
TCANS     : FFELSVEEKEKYANDQTLGKIQGYGSKLANNASGQLEWEDYFFHLVYPEDKRDLSIWPQT : 163
                6         r

                     *       200         *       220         *       240
AT1G75290 : VTNYFAGYYLPTLVQLEPGLTS--------------PPRDKVKIFGDGNVKVE------- : 190
AT1G75280 : VTGCFGGYYLPTLVQFEPGLTS--------------PPRDKVTILGDGNAK--------- : 188
AT1G75300 : VSNCSAGFYLRTLLQFESGLISHTRDKAIIFGDKNVPPRDKVTILGDGNAK--------- : 202
AT1G19540 : VSGCFAGLFVPCLGQCHLRLRS--------------PPRDKVSIYDTGNGK--------- : 187
AT4G39230 : SCNFFAGYFLPTLAQ--PGATS--------------APRDKVIVLGDGNPK--------- : 185
AT4G34540 : CCGLFMRVLLPSLVQ--PGLQS--------------PPTDKVTVFGDGNVK--------- : 183
AT4G13660 : VGACFAAYFGGNLSQMGT----------------LFPPKNKVDIYGDGNVK--------- : 196
AT1G32100 : VGACFAAYFAGNLSQMVT----------------LLPPKEKVNIYGDGNVK--------- : 196
TcLAR     : CCNSIAAWPYHDNTHPADVL----------------PPLDRFKIYGDGTVK--------- : 201
VvLAR     : CCNSIASWPYYNNIHPSEVL----------------PPTDFFQIYGDGNVK--------- : 192
LcLAR1-1  : CCNSIASWPYHDNCHPSKVP----------------PPVDQFLIYGDGTVK--------- : 194
LCLAR1-2  : CCNSIASWPYHDNCHPSKVP----------------PPVDQFLIYGDGSVK--------- : 194
MtLAR     : CCNSIASWPYYDNCHPSQLP----------------PPLDQLHIYGHGNVK--------- : 195
DuLAR     : CCNSIASWPYYDNCHPSQVP----------------PPMDQFQIYGDGNTK--------- : 194
MtDFR     : VSKTLAEQEAWKFAKEHNMDFIT-----------IIPPLVVGPFLIPTMPPS-------- : 205
AtDFR     : VSKTLAEKAAWDFAEEKGLDFIS-----------IIPTLVVGPFITTSMPPS-------- : 205
ZmA1      : VSKTLAEKAALAYAAEHGLDLVT-----------IIPTLVVGPFISASMPPS-------- : 210
AtBAN     : ISKVLAEKTAWEFAKENKINLVT-----------VIPALIAGNSLLSDPPSS-------- : 211
LcANR     : ASKTLAEKAAWKFAEENHIDLIT-----------VIPSLITGPSLTPDIPSS-------- : 210
TcANR     : ASKTLAEKAAWKFAQENNIDLIT-----------VIPSLMTGPSLTPDVPSS-------- : 209
VvANR     : ASKTLAEKAAWKFAEENNIDLIT-----------VIPTLMAGSSLTSDVPSS-------- : 210
CsANR     : LSKTLAEKAAWKFAEENNINLIT-----------VIPTLMAGPSLTADVPSS-------- : 209
ZmA2      : PPDYIAATRDFGRRTRDLASTLLAILSMGL------LGTDRGDALEKALTTTTTRTAADD : 228
PfANS     : PPDYIPATSEYAKQLRALATKILSVLSIG-------LGLEKG-RLEKEVG-------GAE : 212
AtANS     : PSDYIEATSEYAKCLRLLATKVFKALSVG-------LGLEPD-RLEKEVG-------GLE : 206
MtANS     : PADYTKVTSEYAKELRVLASKIMEVLSLE-------LGLEGG-RLEKEAG-------GME : 206
TCANS     : PSDYTEVTSEYARQLRVLASKILSALSLC-------LGLEEG-RLEKEVG-------GLE : 208
```

```
                              *       260         *        280          *       300
AT1G75290 : -YFIAVINKEEDIAAYTIKAVDDPRTLNKTLYINPPNNTLSMNEIVTLWEKKIGKSVEKI : 249
AT1G75280 : ----AVINKEEDIAAYTIKAVDDPRTLNKILYIKPSNNTLSMNEIVTLWEKKIGKSLEKT : 244
AT1G75300 : ----VVINKEEDVAAYMIKAVDDLRTLNKTLYISPPNNILSMNEMVTLWEKKIGKSLEKT : 258
AT1G19540 : ----AIVNTEEDIVAYTLKAVDDPRTLNKILYIHPPNYIVSQNDMVGLWEEKIGKTLEKT : 243
AT4G39230 : ----AVFNKEEDIGTYTINAVDDPRTLNKILYIRPPMNTYSFNDLVSLWENKIGKTLERI : 241
AT4G34540 : ----AVFVNDVDVAAFTIKTIDDPRTLNKTLYLSPPGNICSMNDLVELWEGKIEKKLEKT : 239
AT4G13660 : ----VVFVDEDDMAKYTAKTLNDPRTLNKTVYVRPTDNILTQMELVQIWEKLTEKELEKT : 252
AT1G32100 : ----VVFADEDDIAKYTAKTLNDPRTLNKTVNIRPPDNVLTQLELVQIWEKLTGKELEKT : 252
TcLAR     : ----AYFVAGTDIGKFTIMSIEDDRTLNKTVHFQPPSNLLNINEMASLWEEKIGRTLPRV : 257
VvLAR     : ----AYFVAGTDIGKFTMKTVDDVRTLNKSVHFRPSCNCLNINELASVWEKKIGRTLPRV : 248
LcLAR1-1  : ----AYFVDGNDIGKFTMKAIDDIRTRNKNVHFRPPSNCYSINELASLWEKIIGRKIPRA : 250
LCLAR1-2  : ----AYFVDGNDIGKFTMKAIDDIRTRNKNVHFRPPSNCYSINELASLWEKIIGRKIPRA : 250
MtLAR     : ----AYFVDGYDIGKFTMKVVDDERTINKSVHFRPSTNCYSMNELASLWENKIARKIPRA : 251
DuLAR     : ----AYFIDGNDIGKFTMKTIDDIRTLNKNVHFRPSSNCYSINELASLWEKKIGRTLPRF : 250
MtDFR     : ---LITALSPITGNEAHYSIIKQGQ-------FVHLDDLCEAHIFLFEHMEVEGRYLCSA : 255
AtDFR     : ---LITALSPITRNEAHYSIIRQGQ-------YVHLDDLCNAHIFLYEQAAAKGRYICSS : 255
ZmA1      : ---LITALALITGNAPHYSILKQVQ-------LIHLDDLCDAEIFLFENPAAAGRYVCSS : 260
AtBAN     : ---LSLSMSFITGKEMHVTGLKEMQKLSGSISFVHVDDLARAHLFLAEKETASGRYICCA : 268
LcANR     : ---VGLATSLITGNDFLINAMKGMQLLSGSISITHVEDVCRAHIFVAEKQSASGRYICCA : 267
TcANR     : ---IGLATSLLSGNEFLVNALKGMQMLSGSISITHVEDVCRAHVFLAEKESASGRYICCA : 266
VvANR     : ---IGLAMSLITGNEFLINGMKGMQMLSGSVSIAHVEDVCQAHIFVAEKESASGRYICCA : 267
CsANR     : ---IGLAMSLITGNEFLINGLKGMQMLSGSISISHVEDVCRAHVFVAEKESASGRYICCA : 266
ZmA2      : DLLLQLKINYYPRCPQPELAVGVEAHTDVSALSFILHNGVPGLQVLHGARWVTARHEPGT : 288
PfANS     : DLIVQMKINFYPKCPQPELALGWEAHTDVSALTFILHNMVPGLQLFYEDKWVTAKCVPNS : 272
AtANS     : ELLLQMKINYYPKCPQPELALGVEAHTDVSALTFILHNMVPGLQLFYEGKWVTAKCVPDS : 266
MtANS     : ELLLQMKINYYPICPQPELALGVEAHTDVSSLTFLLHNMVPGLQLFYEGKWVTAKCVPDS : 266
TCANS     : ELLLQMKINYYPKCPQPELALGVEAHTDVSALTFILHNMVPGLQLFYEGKWITAKCVPNS : 268
                                     6                                4

                              *       320         *        340          *       360
AT1G75290 : YMSEE-QIFKSIQESPVPFNVLLSINHAVFVKGDQTNFTIEPSFGFEASELYP--DIKYT : 306
AT1G75280 : HLPEE-QLLKSIQESPIPINVVLSINHAVFVNGD-TNISIEPSFGVEASELYP--DVKYT : 300
AT1G75300 : HISEE-QILKSIQ---VPIDVFKSINHAVFVKGDQTSFTIEPWFGEEASVLYP--DVKYT : 312
AT1G19540 : YVSEE-ELLKTIQESKPPMDFLVGLIHTILVKSDFTSFTIDPSFGVEASELYP--EVKYT : 300
AT4G39230 : YVPEE-QLLKQIIESSPPLNVMLSLCHCVFVKGGHTSFEIEPSFGVEASELYP--DVKYT : 298
AT4G34540 : FATEN-QLLKKIKETPYPDNMEMVFIYSVFIKGDHTYFDIESCGGVNGTELYP--DVKYM : 296
AT4G13660 : YVSGN-DFLADIEDKEISHQAGLGHFYHIYYEGCLTDHEVGD--DEEATKLYP--DVKYK : 307
AT1G32100 : NIAAQ-DFLANIEQMEIPHQAGIGHFYHIFYEGCLTDHEVGE--DEEASSLYP--DVKYK : 307
TcLAR     : TITEE-DLLQMAKEMRIPQSVVAALTHDIFINGCQINFSLDKPTDVEVCSLYP--DTPFR : 314
VvLAR     : TVTED-DLLAAAGENIIPQSVVAAFTHDIFIKGCQVNFSIDGPEDVEVTTLYP--EDSFR : 305
LcLAR1-1  : TVSAE-DLLAAAAENCIPRSIVAAFTHDIFINGCQINFSIEGPNDIEIGTLYP--DEKFR : 307
LCLAR1-2  : TVSAE-DLLAAAAENCIPRSIVAAFTHDIFINGCQINFSIEGPNDIEIGTLYP--DEKFR : 307
MtLAR     : IVSED-DLLGIAAENCIPESVVASITHDIFINGCQVNFKIDGIHDVEISTLYP--GESFR : 308
DuLAR     : TVTAD-KLLAHAAENIIPESIVSSFTHDIFINGCQVNFSIDEHSDVEIDTLYP--DEKFR : 307
MtDFR     : CEANI-HDIAKLINTKYPEYNIPTKFNNIPDELELVRFSSKKIKDLGFEFKYS-LEDMYT : 313
AtDFR     : HDATI-LTISKFLRPKYPEYNVPSTFEGVDENLKSIEFSSKKLTDMGFNFKYS-LEEMFI : 313
ZmA1      : HDVTI-HGLAAMLRDRYPEYDVPQRFPGIQDDLQPVRFSSKKLQDLGFTFRYKTLEDMFD : 319
AtBAN     : YNTSV-PEIADFLIQRYPKYNVLSEFEEGLSIPK-LTLSSQKLINEGFRFEYG-INEMYD : 325
LcANR     : HNTSV-PELAKFLNKRYPQYKVSTEFNDFPAKAK-LIISPEKLIKEGFSFKYG-VEEIFD : 324
TcANR     : VNSSV-PELAKFLNQRYPEFKVPTDFGDFPSKAK-LIISSDKLINEGFSFKFG-IGEIYD : 323
VvANR     : ANTSV-PELAKFLSKRYPQYKVPTDFGDFPPKSK-LIISSEKLVKEGFSFKYG-IEEIYD : 324
CsANR     : VSTSV-PELAKFLNKRYPEYNVPTDFGDFPSKAK-LILSSEKLTKEGFSFKYG-IEEIYD : 323
ZmA2      : IIVHVGDALEILSNGRYTSVLHRGLVNREAVRISWVVFCEPPPDSVLLHPLPELVTEGHP : 348
PfANS     : IIMHIGDTLEILSNGKYKSILHRGLVNKEKVRISWAVFCEPPKEKIVLQPLPETVSEVEP : 332
AtANS     : IVMHIGDTLEILSNGKYKSILHRGLVNKEKVRISWAVFCEPPKDKIVLKPLPEMVSVESP : 326
MtANS     : ILMHIGDTIEILSNGKYKSILHRGLVNKEKVRISWAVFCEPPKEKIILKPLPELVTEKEP : 326
TCANS     : IIMHIGDTVEILSNGKYKSILHRGLVNKEKVRISWAVFCEPPKEKIILKPLPETVSETEP : 328
```

```
                          *        380         *        400         *        420
AT1G75290 : SIDEYLSYFALGTSLNT------------------------------------------------- : 323
AT1G75280 : SVDEYLSYFA-------------------------------------------------------- : 310
AT1G75300 : SIDEYLSQFT-------------------------------------------------------- : 322
AT1G19540 : SVDEFLNRFI-------------------------------------------------------- : 310
AT4G39230 : TVDEILNQYV-------------------------------------------------------- : 308
AT4G34540 : TVSEFLDTLL-------------------------------------------------------- : 306
AT4G13660 : RMDEYLKIFV-------------------------------------------------------- : 317
AT1G32100 : RMDDYLRMFL-------------------------------------------------------- : 317
TcLAR     : TINECFEDFAKKIIDNAKAVS-------------------------------KPAASNNAI : 344
VvLAR     : TVEECFGEYIVKIEE-------------------------------------KQPTADSAI : 329
LcLAR1-1  : CLEECFKDFVPMTHDMNVHVG------------------------------------TTEIN : 333
LCLAR1-2  : CLEECFKDFVPMTHDMNVHVG------------------------------------TTEIK : 333
MtLAR     : SLEDCFESFVAMAADK-IHKG------------------------------------ENGVT : 333
DuLAR     : SLDDCYEDFVPMVHDK-IHAGKSGEIKIKDGKPLVQTGTIEEINKDIKTLVETQPNEEIK : 366
MtDFR     : EAIDTCIEKGLLPK--------FVKSTNK---------------------------------- : 334
AtDFR     : ESIETCRQKGFLPVSLSYQSISEIKTKNENIDVKTGDGLTDGMKPCNKTETGITGERTDA : 373
ZmA1      : AAIRTCQEKGLIPL--------ATAAGGDGFASVRAPGETEATIGA-------------- : 357
AtBAN     : QMIEYFESKGLIKAK--------------------------------------------- : 340
LcANR     : QTLEYLKTKGALKN---------------------------------------------- : 338
TcANR     : QTVEYMNAKGLLK----------------------------------------------- : 336
VvANR     : ESVEYFKAKGLLQN---------------------------------------------- : 338
CsANR     : QSVEYFKAKGILKN---------------------------------------------- : 337
ZmA2      : ARFTPRTFKQHLDRKLFKKKQQHKAKAEEEDGGNGDHHRHEPPPQTN------------- : 395
PfANS     : PRFPPRTFAQHLKHKLFRKTDGDLDEKPTY------------------------------ : 362
AtANS     : AKFPPRTFAQHIEHKLFGKEQEELVSEKND------------------------------ : 356
MtANS     : ARFPPRTFAQHIHHKLFRKDEEEKKDDPKK------------------------------ : 356
TCANS     : PLFPPRTFAQHIHHKLFRKTQDGLSN---------------------------------- : 354

                          *
AT1G75290 : ----------------- :   -
AT1G75280 : ----------------- :   -
AT1G75300 : ----------------- :   -
AT1G19540 : ----------------- :   -
AT4G39230 : ----------------- :   -
AT4G34540 : ----------------- :   -
AT4G13660 : ----------------- :   -
AT1G32100 : ----------------- :   -
TcLAR     : FVPTAKPGALPITAICT : 361
VvLAR     : ANTGPVVGMRQVTATCA : 346
LcLAR1-1  : NNRKSLVEVAPITAMG- : 349
LCLAR1-2  : NNRKSLVEVAPITAMG- : 349
MtLAR     : GGTKALVEPVPITASC- : 349
DuLAR     : KDMKALVEAVPISAMG- : 382
MtDFR     : ----------------- :   -
AtDFR     : PMLAQQMCA-------- : 382
ZmA1      : ----------------- :   -
AtBAN     : ----------------- :   -
LcANR     : ----------------- :   -
TcANR     : ----------------- :   -
VvANR     : ----------------- :   -
CsANR     : ----------------- :   -
ZmA2      : ----------------- :   -
PfANS     : ----------------- :   -
AtANS     : ----------------- :   -
MtANS     : ----------------- :   -
TCANS     : ----------------- :   -
```

Supplemental Figure S1. Multiple sequence alignment of the LAR, ANS and ANR proteins as well as related IFR and DFR proteins of the RED superfamily. The protein sequences were aligned using the default parameters of ClustalX. The alignment includes LAR, ANR and ANS proteins from cacao, as well as from other species whose enzymatic activities has been shown in previous publications. Also included are 8 IFR like proteins from Arabidopsis. IFR-like proteins from Arabidopsis are labeled with their locus numbers. All the other proteins are labeled by the species they come from followed by their catalytic activities. The species represented and their GenBank accession numbers in the LAR group are *Medicago truncatula* (MtLAR, CAI56327), *Vitis vinifera* (VvLAR1, CAI26309), *Desmodium uncinatum* (DuLAR, CAD79341) and *Lotus corniculatus* (LcLAR1-1, ABC71324; LcLAR1-2, ABC71325); in the DFR group are *Zea mays* (ZmA1, CAA28734), *Arabidopsis thaliana* (AtDFR, NP_199094) and *Medicago truncatula* (MtDFR, AAR27014); in the ANR group are *Arabidopsis thaliana* (BAN, NP_176365), *Medicago truncatula* (MtANR, AAN77735), *Vitis vinifera* (VvANR, CAD91911), *Camellia sinensis* (CsANR, AAT68773) and *Lotus corniculatus* (LcANR, ABC71337); and in the ANS group are *Perilla frutescens* (PfANS, O04274), *Arabidopsis thaliana* (AtANS, Q96323), *Zea mays* (ZmA2, CAA39022) and *Medicago truncatula* (MtANS, ABU40983).

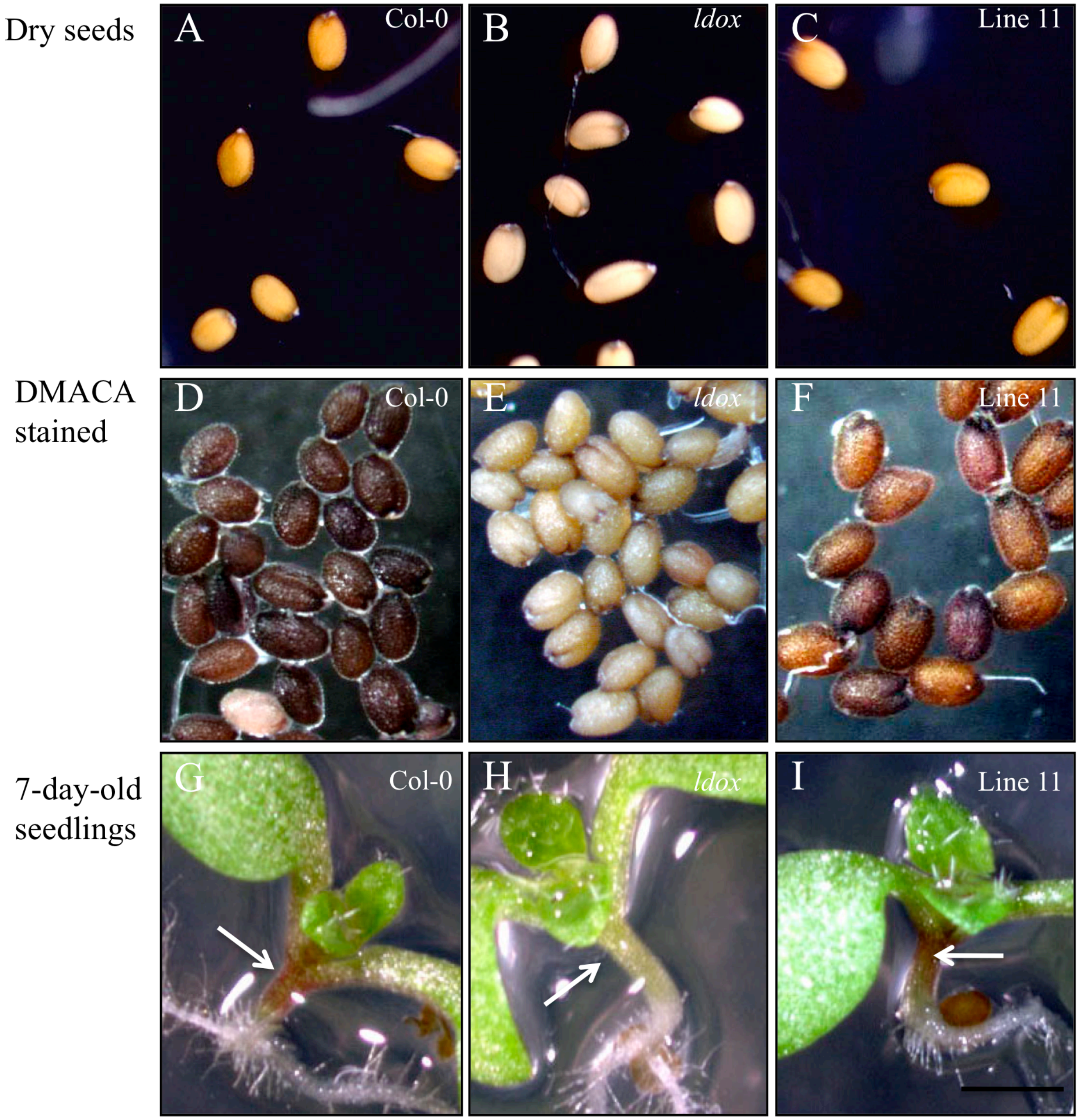

Supplemental Figure S2. Complementation of the PA- and anthocyanin-deficient *ans* (*ldox)* mutant phenotype by constitutively expressing *TcANS*. Two independent lines showed a similar pattern to Col-0 for PA and anthocyanin accumulation. A, Brown Col-0 wild type seeds. B, Pale yellow *ldox* mutant seeds (SALK_028793). C, Brown *ldox* 35S:TcANS seeds. D to F, PAs were localized by staining the seeds with DMACA, which specifically stains PAs blue. D, Col-0 wild type seeds. E, *ldox* mutant seeds. F, *ldox* 35S:TcANS seeds. G and H, Anthocyanin accumulation in the upper hypocotyls of 7-day-old seedlings. G, Col-O wild type seedling. H, *ldox* mutant seedling. I, *ldox* 35S:TcANS seedling. Arrows in G, H and I point to the upper hypocotyl region. The bar represents 2 mm (A to I).

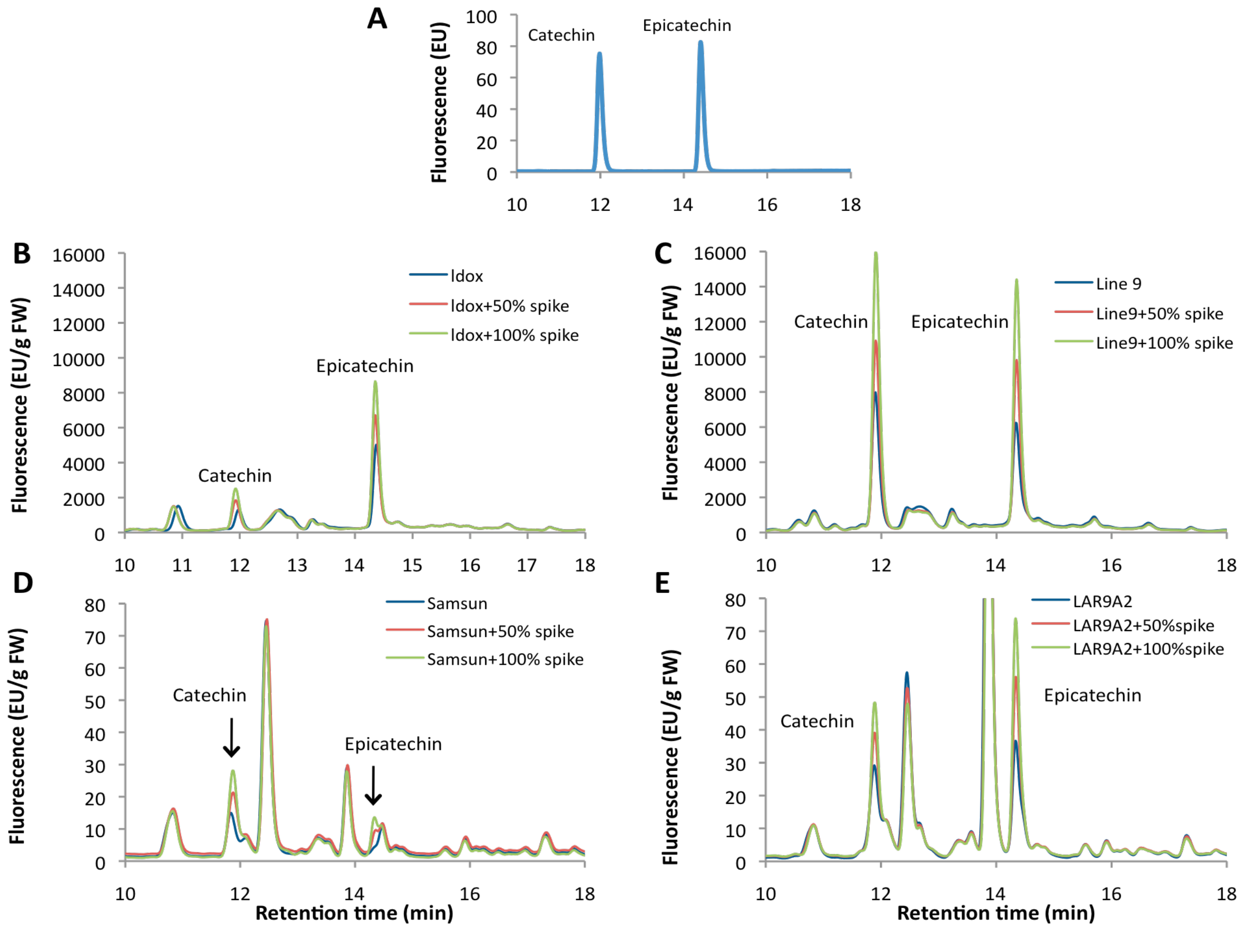

Supplemental Figure S3. HPLC analysis of extracts from transgenic Arabidopsis and tobacco samples with and without spiking with known standards to validate the identity of the catechin and epicatechin peaks. A, Catechin and epicatechin standards used in spiking. B, Young siliques extracts from Arabidopsis *ldox* mutant with and without standard spikes. C, Young siliques extracts from transgenic Arabidopsis 35S:*TcLAR* Line9 with and without standard spikes. D, Flower petal extracts from wildtype tobacco (Samsun) with and without standard spikes. E, Flower petal extracts from transgenic tobacco 35S:*TcLAR*:Samsun Line LAR9A2 with and without standard spikes. FW, fresh weight; EU, emission units.

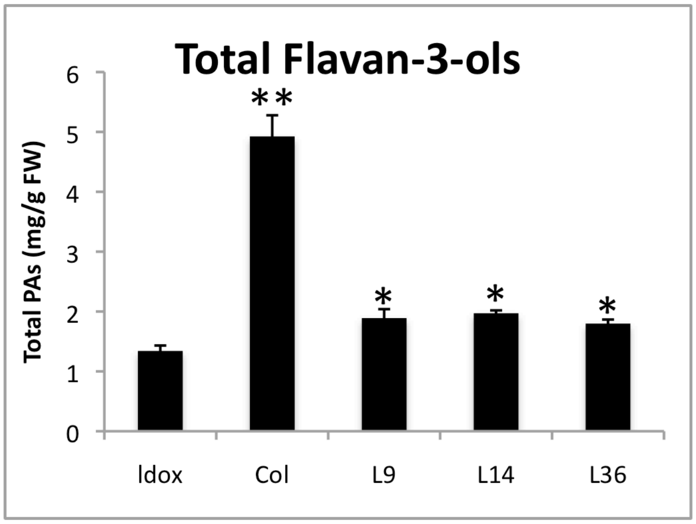

Supplemental Figure S4. Complementation of the PA deficient Arabidopsis *ldox* mutant phenotype by constitutive expression of *TcLAR*. PA levels in mature seeds of three different lines of transgenic plants. Total flavan-3-ols levels were determined by extraction and DMACA reaction with procyanidin B2 as standards. All data are presented as mean values ± SE, n=3. *P < 0.05 versus *ldox*; **P < 0.01 versus *ldox*.
